# Influence of Imidazole Substituent Bulkiness on [CuI(PPh_3_)_2_N] Complexes with TADF Blue Solid-State Emission

**DOI:** 10.1021/acsomega.5c10663

**Published:** 2026-02-19

**Authors:** Carolina Francener, Giliandro Farias, Renê Santos de Amorim, Marcelo Meira Faleiros, Larissa Gomes Franca, Andrew P. Monkman, Adailton J. Bortoluzzi, Teresa Dib Zambon Atvars, Eduard Westphal, Ivan H. Bechtold

**Affiliations:** † 28117 Universidade Federal de Santa Catarina, Department of Chemistry, R. Agronomico Andrei Cristian Ferreira, Florianópolis 88040-900, Brazil; ‡ Universidade de São Paulo, Department of Materials Physics and Mechanics, R. da Reitoria, São Paulo 05508-220, Brazil; § 28132 Universidade Estadual de Campinas, Department of Chemistry, Cidade Universitaria Zeferino Vaz, Campinas, SP 13083-970, Brazil; ∥ 2152 University of Cambridge, Department of Materials Science and Metallurgy, Trinity Ln, Cambridge CB3 0FS, U.K.; ⊥ 3057 Durham University, Department of Physics, South Rd, Durham DH1 3LE, U.K.; # Universidade Federal de Santa Catarina, Department of Physics, R. Agronomico Andrei Cristian Ferreira, Florianópolis 88040-900, Brazil

## Abstract

Copper­(I) heteroleptic complexes with phosphine- and nitrogen-containing ligands are well-known thermally activated delayed fluorescence (TADF) emitters and are of interest for OLED applications due to their cost-effectiveness. In this work, we report the design and synthesis of a series of novel Cu­(I) complexes featuring triphenylphosphine- and imidazole-based ligands functionalized with varying alkyl chains. This allowed for the evaluation of purely monodentate ligand systems and the impact of the ligand bulkiness on the photophysical properties. Time-resolved spectroscopy, combined with temperature-dependent measurements, revealed blue solid-state emission and confirmed the TADF behavior across the series. Room-temperature lifetimes reached as low as 0.5 μs, facilitated by small singlet–triplet energy gaps Δ*E*_(S1–T1)_ between 110 and 60 meV. Bulky ligands led to the smallest S–T gaps. A decrease in phosphorescence lifetimes with increasing alkyl chain size suggested enhanced spin–orbit coupling (SOC), attributed to a more tetrahedral coordination environment. Quantum chemical calculations revealed that the imidazole moiety does not participate directly in the frontier molecular orbitals, indicating that the alkyl chains primarily influence the emission by modulating the geometry around the Cu­(I) center.

## Graphical abstract

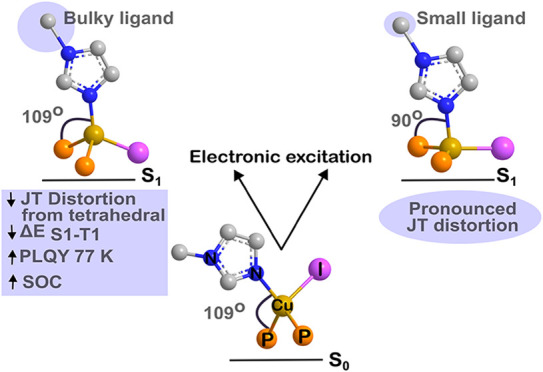

## Introduction

The search for novel luminescent materials viable for the commercial application of organic light-emitting diodes (OLEDs) has been a driving force of research in the past decades.^1–3^ Thermally activated delayed fluorescence (TADF) appears as an alternative with the potential of using 100% of the excited states generated by electroexcitation, without the need for scarce, high-cost metals found in efficient phosphorescence emitters, like Pt and Ir.^4–6^ TADF is a triplet harvesting process where there is the conversion of non- or low radiative triplet states into emissive singlet states.^7^ One of the main prerequisites for TADF is an energy gap between S_1_ and T_1_ (Δ*E*_(S1–T1)_) inferior to 1500 cm^–1^ (0.18 eV).^8^ A driving force to obtain close-lying singlet–triplet states is the physical separation and small overlap of the frontier orbitals, which minimizes the electron exchange energy.^9^ Contrastingly, the degree of HOMO–LUMO overlap is directly related to the oscillator strength between S_1_ and S_0_ states and, inversely, to emission lifetime.^10^ Therefore, a balance between these two factors through molecular design is imperative. The development of stable, long-lasting blue-emitting OLEDs is hindered due to intrinsic and extrinsic factors related to the nature of the emitters and has been the focus of several studies.^11,12^

McMillin and co-workers, in 1980, published the first investigation of the emissive properties of a Cu­(I) coordination compound, [Cu­(dmp)_2_]+, unravelling the emission of such molecules through TADF, which has led the way for decades of study on copper­(I) complexes.^13^ Copper­(I) coordination compounds tend to have closely lying singlet and triplet states, allowing highly efficient TADF processes and low synthesis and processing costs, thereby enabling affordable OLED devices.^14–16^

After electronic excitation, the tetracoordinated complexes with tetrahedral geometry migrate to a Franck–Condon (FC) S_n_-metal–ligand charge-transfer (MLCT) excited state, featuring an oxidized d^9^ Cu center. From here, the electrons can relax through internal conversion to ^1^MLCT S_1_ or intersystem crossing to ^3^MLCT T_1_. In both the singlet and triplet manifolds, the complexes will tend to go through a flattening to a planar square geometry because of the pseudo-Jahn–Teller (JT) effect caused by the uneven electron distribution in the excited copper core.^17^ The distorted ^1^MLCT and ^3^MLCT (MLCT_flattened_) states lie closely in energy and can undergo intersystem crossing (ISC) and reverse intersystem crossing (rISC) between them. In the thermally induced rISC process, delayed fluorescence can be observed. The excited-state distortion favors nonradiative processes, thereby decreasing the emission quantum yield. Moreover, ISC and rISC are nonradiative processes forbidden by the spin multiplicity rule that can be relaxed by the spin–orbit coupling (SOC) mechanism. MLCT S_1_ and MLCT T_1_ usually have the same electronic configurations (d_1_→π*) and, therefore, a small, though not negligible, exchange is expected between them.^18^ However, MLCT T_1_ can interact with MLCT S_2_ and MLCT S_3_, whose characters involve a separate d orbital (d_2_), thereby increasing the coupling between the singlet and triplet manifolds. The magnitude of the SOC depends on how energetically close d_1_ and d_2_ orbitals are. In a tetrahedral geometry, d orbitals intrinsically lie closer together, whereas the energy gap between them is larger in a square planar configuration.^19,20^ Therefore, molecular design employing bulky ligands is a viable approach to hinder the deformation of the tetrahedral arrangement,^21^ improving the coupling between the singlet and triplet manifolds. Steric hindrance caused by the ligands also aids in reducing the nonradiative decay, succeeded by the JT effect.^22,23^

Unlike organometallics with an Ir­(III) center with a high SOC constant (ξ_soc_ > 3000 cm^–1^),^24^ copper is a light metal, and the Cu­(I) nucleus has an SOC constant of only 857 cm^–1^, which leads to slow rates of ISC.^25^ For Cu­(I) materials to be considered for OLED devices, it is necessary to develop molecular designs that simultaneously yield a high quantum yield (QY) and short radiative lifetimes. In this respect, the coordination of Cu­(I) with halides, such as iodine (ξ = 5069 cm^–1^), can induce faster ISC between singlet and triplet states, as well as promote an efficient and short-lived phosphorescence decay.^26,27^ This dual emission leads to increased QY while maintaining the color purity of devices, for materials with a small singlet–triplet energy gap. Phosphine-derived ligands pose themselves as promising ligands for Cu­(I) complexes due to their spatial steric hindrance effect, along with a strong coordination ability with the soft acidic Cu­(I) metal.^28^ Moreover, recent studies by us have led to the conclusion that a combination of P-containing ligands and N-heterocyclic compounds provides complexes with closely lying S–T states and efficient emissions.^29–33^

In this work, we investigate the optical properties of a newly designed series of Cu­(I) complexes and examine how the bulkiness of imidazolium ligands influences their behavior. Each complex consists of a Cu­(I) center coordinated to an iodine atom, a triphenylphosphine (PPh_3_) group, and an imidazole-based ligand bearing different alkyl substituents. The compounds were chemically characterized. The thermal stability and electrochemical performance were studied. Their spectroscopic features were explored using steady-state and time-resolved techniques, supported by theoretical calculations to provide deeper insight into structure–property relationships.

## Results and Discussion

### Synthesis and Characterization

The designed copper complexes were synthesized by using an established method without the need for an inert atmosphere or anhydrous solvents. Copper­(I) iodide (CuI) was stirred with PPh_3_ and the respective alkylated imidazole in chloroform in a 1:2:1 molar ratio. The materials were purified by crystallization in diethyl ether and obtained with yields above 60%. The synthetic route for the complexes is shown in Scheme 1. Detailed experimental procedures and analytical data are provided in the Supporting Information.

**Scheme 1:**
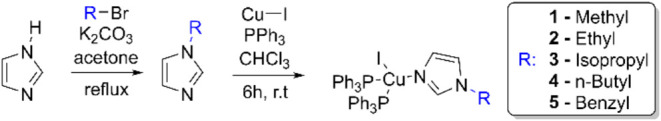
Synthetic Scheme and Reaction Conditions for Compounds **1–5**

For all products, the ratio of signals in the ^1^H NMR spectra to the elemental analysis data is consistent with the proposed mononuclear structure for the Cu­(I) complexes (see Supporting Information for complete characterization). Additionally, for complex **3**, a single crystal was successfully obtained through the solvent diffusion method employing chloroform and diethyl ether as solvents and antisolvents, respectively (CCDC 2442778). XRD analysis of the single crystal proved the mononuclear structure of the compound (Figure 1). Therefore, the similarity of the ^1^H NMR and elemental analysis results for all products, combined with the single-crystal XRD data, is a strong indication that all complexes have a mononuclear structure. For compound **3**, the copper atom acquires a distorted tetrahedral configuration with a P–Cu–P angle of 126.5°. Cu–N, average Cu–P, and Cu–I bond lengths are 2.042, 2.227, and 2.706 Å, respectively (Table S1).

**Figure 1:**
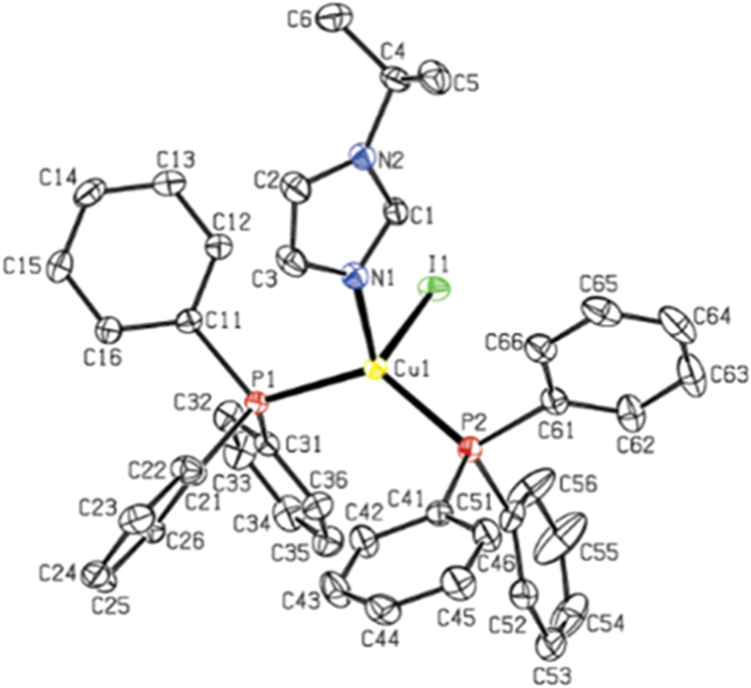
ORTEP plots (thermal ellipsoids with 50% probability) of the molecular structure of **3**. Hydrogen atoms were omitted for clarity.

Thermogravimetric analysis was performed to investigate the thermal stability of the complexes (Figure 2a). Compounds **1–4** are stable upon heating to 125 °C, presenting less than 0.5% weight loss. Complex **5** showed mass loss around 100 °C due to water molecules in its crystal structure, corroborating with elemental analysis data (see Supporting Information). Despite being unsuitable for thermal deposition into devices, the complexes high solubility and stability in solution allow for solution processing of films.^34^ Cyclic voltammograms (Figure 2b) show an oxidation peak around 0.53 to 0.65 V for all compounds, related to the Cu^1+^ to Cu^2+^ conversion.^35,36^ **5** shows the smallest oxidation potential, potentially due to π–π interactions between the benzyl rings, which flattens the molecule and destabilize the metal core. Given the irreversible nature of the photochemical process, only the HOMO energy could be extracted. The potentials were −5.36, −5.32, −5.31, −5.32, and −5.34 for **1** to **5**, respectively. Optical techniques were used to determine the band gap energies of the compounds (Table 1).

**Figure 2:**
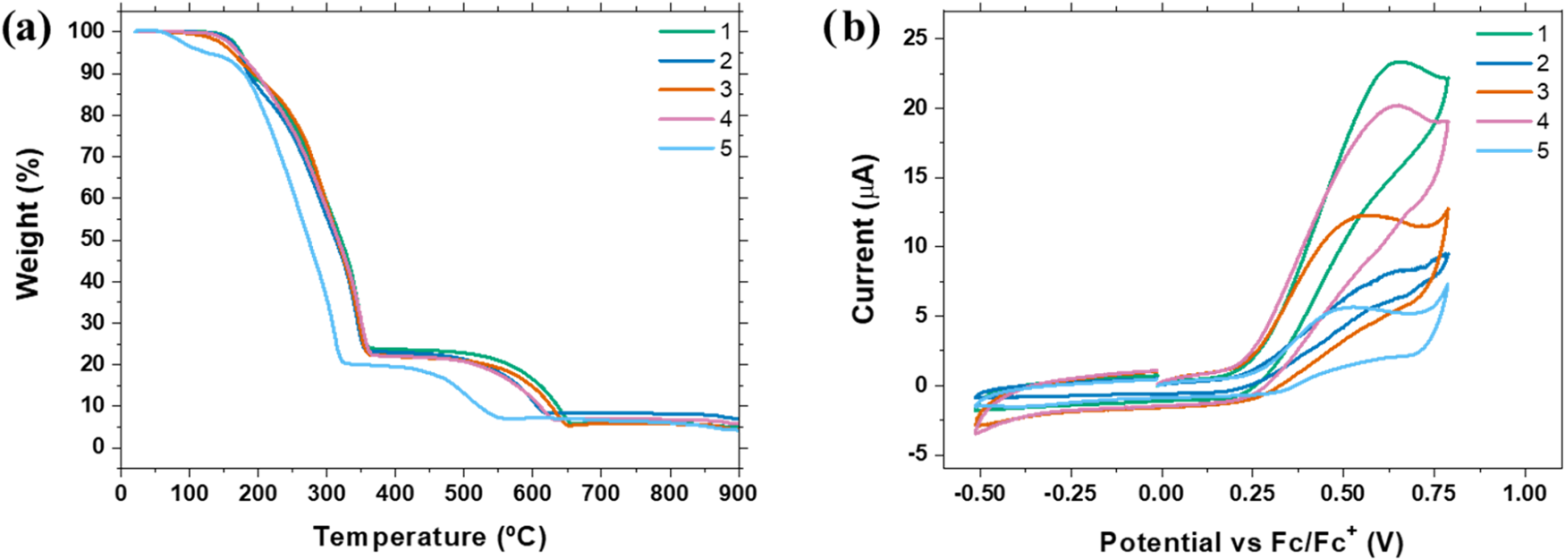
(a) Thermogravimetry of complexes **1–5.** (b) Cyclic voltammograms of **1–5** in 2 × 10^–3^ dichloromethane solution vs (Fc/Fc^+^) couple. nBu_4_NPF_6_ was used as a supporting electrolyte.

**Table 1:** Summary of Photophysical Results

		**1**	**2**	**3**	**4**	**5**
**298 K**	λ_Abs_/nm (ε/L·cm^–1^·mol^–1^)^a^	362 (−)	360 (11299)	356 (10586)	353 (16089)	321 (9783)
	λ_Em_/nm	472	462	473	461	470
	*E*_gap solution_ (eV)	3.83	3.83	3.83	3.83	3.83
	*E*_gap solid_ (eV)	3.75	3.76	3.79	3.78	3.80
	*τ*_298 K_/μs^b^	6	3	1	4	0.5
	Φ_298 K_/%	21.4	7.4	3.4	1.7	2.6
	*k*_r_/s^–1^ ^c^	3.5 × 10^4^	2.4 × 10^4^	3.4 × 10^4^	4.2 × 10^3^	5.2 × 10^4^
	*k*_nr_/s^–1^ ^d^	1.5 × 10^5^	3.1 × 10^5^	9.6 × 10^5^	2.4 × 10^5^	1.9 × 10^6^
**77 K**	τ_77 K_/μs	281	265	203	122	181
	Φ_77 K_/%	59.9	18.7	20.8	58.9	30.2
	*k*_r_/s^–1^ ^c^	2.1 × 10^3^	7.0 × 10^2^	1.0 × 10^3^	4.8 × 10^3^	1.7 × 10^3^
	*k*_nr_/s^–1,^ ^d^	1.4 × 10^3^	3.1 × 10^4^	3.9 × 10^3^	3.4 × 10^3^	1.8 × 10^4^
	*k*_S1_/s^–1^	3.5 × 10^6^	1.8 × 10^7^	3.6 × 10^6^	2.8 × 10^6^	6.5 × 10^6^
	*k*_T1_/s^–1^	2.6 × 10^3^	4.0 × 10^3^	5.2 × 10^3^	8.8 × 10^3^	6.3 × 10^3^
	Δ*E*_(S1–T1)_/eV (in cm^–1^)	0.09 (749)	0.08 (698)	0.11 (866)	0.07 (555)	0.06 (510)
	*k*_TADF_/s^–1^ ^e^	1.6 × 10^5^	3.3 × 10^5^	9.9 × 10^5^	2.4 × 10^5^	2.0 × 10^6^

a Epsilon measured in the chloroform solution.

b Average lifetime: $\tau =\frac{\sum_{i}\tau_{i}^{2}A_{i}}{\sum_{i}\tau_{i}A_{i}}$

c $k_{r}=\frac{\phi }{\tau }$

d $k_{\mathrm{nr}}=\frac{\left(1‐\phi \right)}{\tau }$

e $k_{TADF}=\frac{1}{\tau \;\left(298\;K\right)}-k_{r\;\left(77\;K\right)}$

### Optical Properties

UV–visible and photoluminescence (PL) spectra were recorded in a chloroform solution. Complexes **1**, **3**, and **5** were used as representatives of the whole series and exhibit near-identical solution-state behavior. Absorption spectra (Figure 3a, dashed curves) showed a strong band centered at 260 nm, assigned to an intraligand (IL) π–π* character with contributions from both PPh_3_ and imidazole ligands.^37,38^ A shoulder was observed at 300 nm, which shows a negligible ligand contribution (Figure S25) and is attributed to a CT transition. DFT calculations elucidated the charge transfer character of the transition. More specifically, charge is transferred from the d orbitals of copper and the p orbitals of iodine to the π* orbitals in the PPh_3_ moiety. Therefore, the transition is assigned as (M+X)­LCT, as is the case for other Cu­(I) complexes with halides as ligands.^16,39^ The Stokes shift of 0.69 eV between CT absorption and emission indicates a notable degree of structural reorganization in the excited state. PL was measured under excitation at the charge transfer transition (300 nm; Figure 3a, solid curves). The curves exhibited a broad profile with a 360 nm peak and were identical.

**Figure 3:**
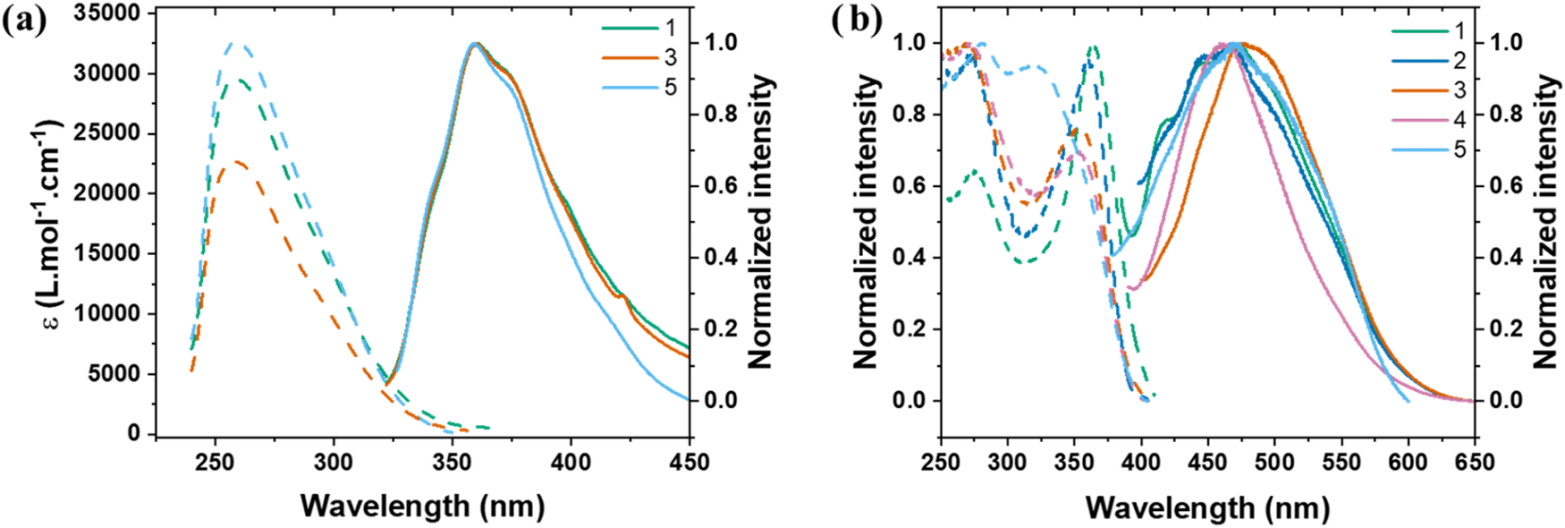
(a) Absorption (dashed) and emission (solid) spectra for complexes **1**, **3**, and **5** in 4 × 10^–5^ mol L^–1^ CHCl_3_ solution. (b) Excitation (dashed) and emission (solid) spectra of complexes **1–5** in powder form.

Excitation and PL were subsequently measured in the powder form. On the excitation profile (Figure 3a, dashed curves), the same IL and MLCT transitions were present; the lower energy CT band was blue-shifted with the increased alkyl chain. The shift was more noticeable for compound **5**. All complexes presented blue emission in the solid state (Figure 3b, solid curves). PL curves displayed higher vibronic character compared to emission in solution, despite the CT character, with peaks at 472, 462, 473, 461, and 470 nm for complexes **1** to **5**, respectively. Emission in solution was unaffected by the bulkiness of the imidazole ligand; however, the varied alkyl chains caused shifts in excitation and PL in the solid state, hinting at electronic effects that affect the energy levels of the CT states.

Absolute photoluminescence quantum yields (PLQY) of the powder samples were determined (Table 1). At room temperature, complex **1** exhibited a quantum efficiency of 21%, whereas the remaining series showed values below 8%, decreasing with the increasing number of Csp^3^ atoms; complex **4** displayed the lowest efficiency at 1.7%. At 77 K, complexes **1** and **4** achieved the highest PLQY values, 59.9 and 58.9%, respectively, suggesting that the voluminous *n*-butyl chain in complex **4** may suppress excited-state distortion, thereby enhancing the system’s quantum efficiency. These results indicate that PLQY at room temperature is limited by nonradiative decay pathways arising from alkyl chain vibrations.

Complex **1** emission at room temperature had a lifetime of τ (298 K) = 6 μs, which increased to τ (77 K) = 281 μs at liquid nitrogen temperature. The radiative rate decreased 10-fold as the temperature was lowered, from *k*_r_ (298 K) = 3.5 × 10^4^ s^–1^ to *k*_r_ (77 K) = 2.1 × 10^3^ s^–1^ (Table 1). Emission at 77 K is attributed to phosphorescence. As the temperature increased, it became thermally activated into a distinct emission process with a higher radiative decay rate and spin-allowed transitions. The extended emission lifetime at RT, on the order of microseconds, pointed to a delayed fluorescence mechanism. This is further discussed in the next section.

### Observation of Thermally Activated Delayed Fluorescence

To deepen the understanding of how the radiative processes are affected by thermal energy and assess the possibility of delayed fluorescence behavior, decay curves of powder emission were collected between 298 and 77 K. The decay curves are displayed in Figures 4a,b, and S26–S29. The fitted lifetimes are listed in Table 1. At RT, the complexes exhibited biexponential decays, with average lifetimes ranging from 6 μs to 500 ns across the series. The decay time increased as the sample was cooled, reaching up to 281 μs. The observation indicated the trapping of electrons in a state with reduced spin allowance as thermal energy is reduced. Moreover, as will be discussed below, the singlet–triplet energy gap Δ*E*_(S1–T1)_ lies below 150 meV for the emitters. Therefore, the long-lived fluorescence, temperature effect, and energetic proximity of the singlet and triplet states lead to the conclusion of a TADF mechanism. The states involved in the radiative transitions have (M+X)­LCT character, as was previously stated. Therefore, at low temperature, emission is predominantly observed from a ^3^(M+X)­LCT state. rISC is thermally activated as the temperature increases, favoring emission from ^1^(M+X)­LCT.

**Figure 4:**
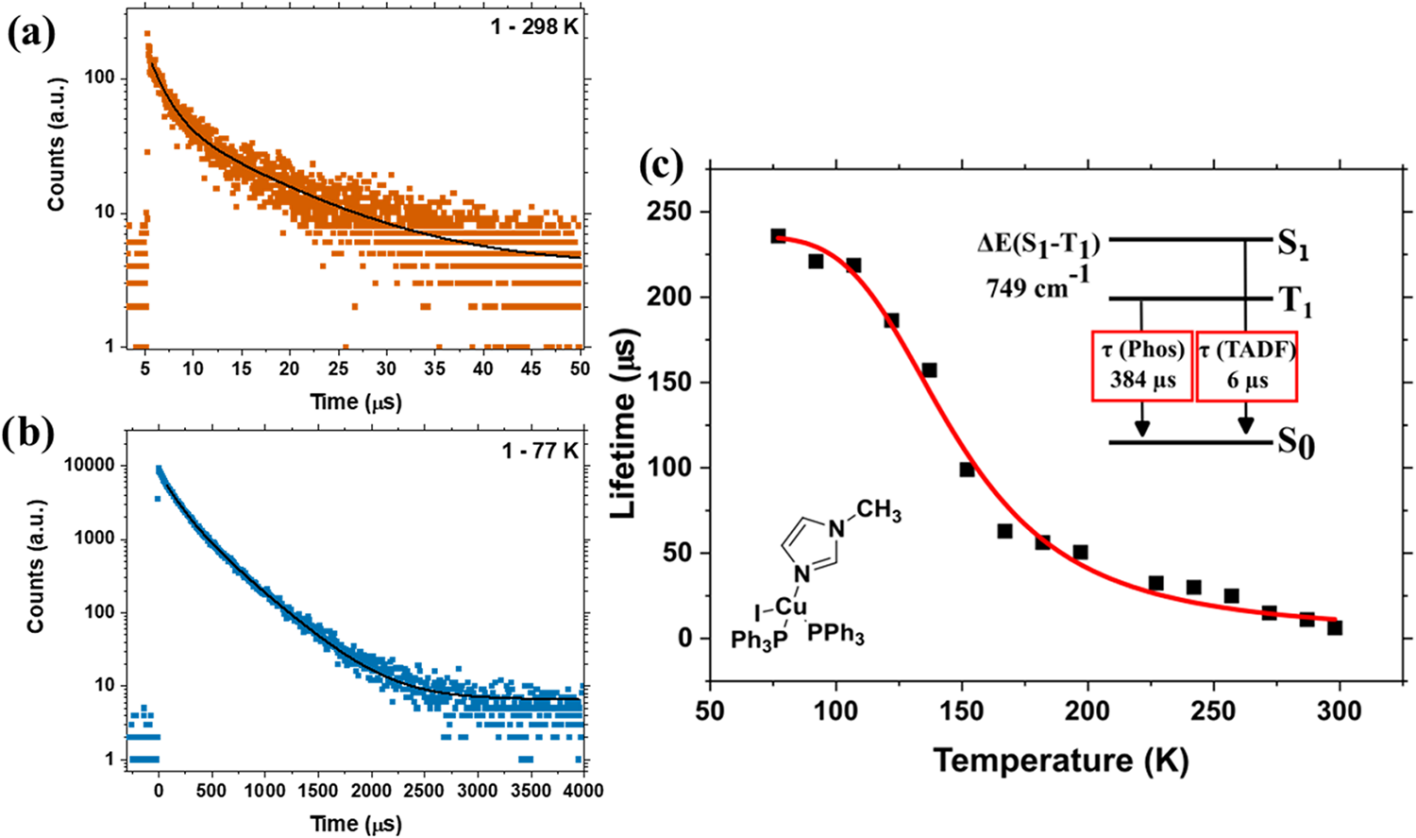
(a) Decay curve of **1** in powder form monitored at 450 nm at RT. (b) Decay curve of **1** in powder form monitored at 450 nm at 77 K. (c) Dependence of the emission lifetime with the temperature for **1**. The red line represents the fitting to eq S1.

The lifetimes as a function of temperature for **1** bear a sigmoidal profile (Figure 4c); however, a lifetime plateau is not observed at 77 K, indicating that rISC occurs even at a low temperature. Curves for **2–5** are found in Figure S30. The widely accepted model for TADF describes the S_1_ and T_1_ states as being in thermal equilibrium, with T_1_ acting as a long-lived exciton reservoir. The emission lifetime depends on the temperature, the radiative decay rate constants of the S_1_ and T_1_ states (*k*_S1_ and *k*_T1_, respectively), and the Δ*E*_(S1–T1)_, as described by the Boltzmann-type equation (eq S1).^40–42^ SOC is enhanced by the heavy-atom effects of Cu and I, thereby contributing to the spin-flip processes. Therefore, an equilibrium between singlet and triplet states is assumed.^43,44^ By fitting this equation to the temperature-dependent decay times, we determined that the prompt fluorescence lifetimes (τ_S1_ = *k*_S1_^–1^) ranged between 55 and 350 ns across the series. In contrast, the phosphorescence lifetimes (τ_T1_) ranged from 110 to 390 μs. Despite the relatively long τ_S1_, negligible spontaneous fluorescence was observed, indicating that ISC outcompeted prompt fluorescence. The Δ*E*_(S1–T1)_ values ranged from 110 to 60 meV, depending on alkyl chain bulkiness.

The radiative and nonradiative decay rate constants (*k*_r_ and *k*_nr_, respectively) at 298 and 77 K were calculated, and the values are displayed in Table 1. The radiative decay rate constant of the series was overall in the same order of magnitude as Cu­(I) complexes with more rigid structures.^45–47^ Heteroleptic cationic complexes of the type [Cu­(N^N)­(P^P)]^+^, with calcogenated diimine and diphosphine chelating ligands, presented *k*_*r*_ values between 3.6 × 10^4^ and 10 × 10^4^ s^–1^,^30^ whereas a different series with a [Cu­(N^N)­(P)­(I)] structure showed radiative constant in the order of 10^4^ s^–1^.^29^

By applying the output of fitting of eq S1, Φ_298 K_ and Φ_77 K_ data into eq S2 and S3, we could visualize the contribution of S_1_ and T_1_ states to the total intensity (*I_tot_)* of emission in dependence on the temperature.^48^ The curves for **1–5** (Figure S31) illustrated how phosphorescence is the prevailing photophysical process at low temperatures (*T* < 100 K). Contribution from delayed fluorescence increases as temperatures rise, and at room temperature, decay comes predominantly from S_1_ for all materials, although TADF contribution is affected by alkyl substituent.

To further evaluate the emissive behavior of complexes **1**, **4**, and **5**, time-resolved spectroscopy was conducted at 298 and 80 K. At 298 K (Figure 5, top), early time emission is dominated by fast, high-energy prompt fluorescence (ns range), followed by a red-shifted, weaker phosphorescence (ms range). Intermediate delay curves show a gradual red shift, indicating increased triplet-state contribution. At 80 K (Figure 5, middle), emission intensity in the ns−μs range decreases, consistent with temperature-dependent behavior characteristic of TADF. From the onset energies of prompt fluorescence and phosphorescence at 80 K, where TADF is minimized, the Δ*E*_(S1–T1)_ values for complexes **1**, **4**, and **5** were determined as 80, 100, and 70 meV, respectively, matching values obtained via eq S3 and confirming consistency between both methods. Delayed fluorescence persists at 80 K, implying sufficient thermal energy for the rISC despite the low temperature. At room temperature, the ms emission is red-shifted and longer-lived compared to 80 K, and Δ*E*_(S1–T1)_ values derived from these curves are inconsistent with TADF. This is likely due to emissions coming from low-lying aggregate states. The isoemissive point at 475 nm supports the presence of an additional emitting species (Figure S32). At 80 K, suppressed electron hopping enables observation of monomer phosphorescence only or at an improved percentage. Additionally, the decay profiles (Figure 5, bottom line) further confirmed the persistence of delayed fluorescence emission at 80 K, although at a lower intensity. Low-temperature TADF is possible in the emitters due to the small energy gap between the S and T states. Moreover, theoretical calculations revealed significant spin–orbit coupling, as will be further discussed, further favoring rISC. Despite the reduction in the DF, the PLQY of the powder suffered a significant increase when T was lowered (Table 1). Therefore, it is concluded that structural aspects, such as suppression of molecular vibrations and excited-state distortion, bear a higher impact on the quantum efficiency against the delayed fluorescence.

**Figure 5:**
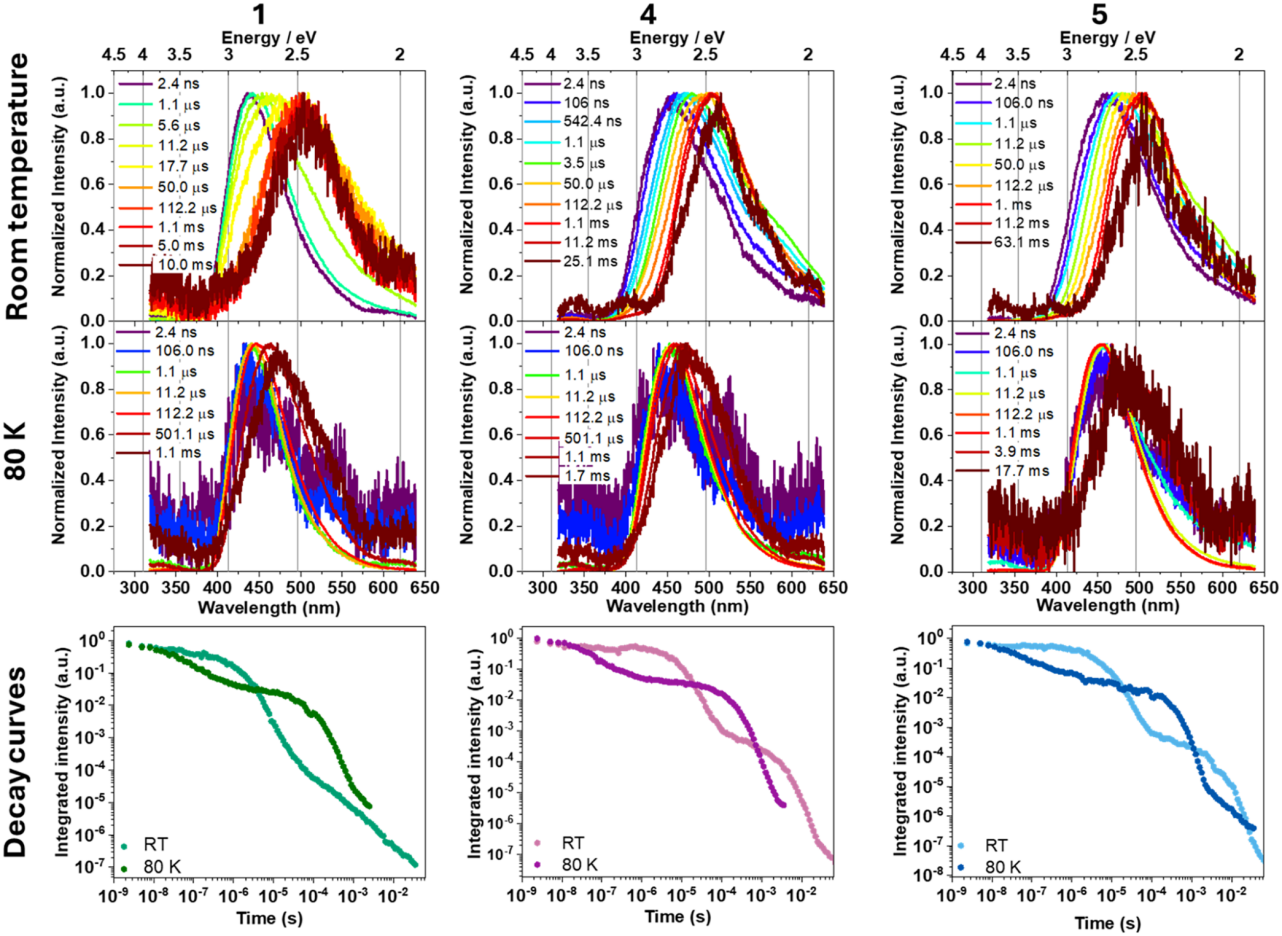
Top line: Time-resolved spectra at room temperature for powder samples under 355 nm excitation for **1, 4**, and **5**. Middle line: Time-resolved emission spectra at 80 K for powder samples under 355 nm excitation. Bottom line: Time-resolved decay curves for powder samples at room temperature and 80 K.

### Impact of Ligand Bulkiness

Overall, the complexes exhibited a similar photophysical behavior in solution, consistent with their minimal structural variation. In dilute CHCl_3_ solution, all compounds showed identical emission profiles and energies. However, in the solid state, both excitation and emission spectra varied with changes in the alkyl chain length on the imidazole ligand. This suggests that the substitution does not significantly alter the molecular energy levels but may influence molecular packing or aggregation. This will be further explored in the theoretical section. As mentioned above, **4**, bearing the longest acyclic chain of the series, exhibited the lowest solid-state QY at RT and showed a significant improvement when T was lowered to 80 K (1.7 to 58.9%), reaching the same efficiency as **1** when error margins are considered. The same behavior is observed when looking at the radiative decay rates, where **4** has the highest *k*_r_ among the series at 80 K (*k*_r (298 K)_ = 4.2 × 10^3^ s^–1^ and *k*_r (77 K)_ = 4.8 × 10^3^ s^–1^). Moreover, **4** displayed the shortest lifetime in the series at 77 K, 122 μs. Analogous behavior is observed for **5**. The satisfactory performance at **4** at frozen temperatures indicated that the elongated ligand attached to the metal core is somehow beneficial to the decay processes; however, at room temperature, enhanced nonradiative deactivation arising from the alkyl chain reduces emission efficiency. Since the predominant decay at 77 K comes from phosphorescence, T_1_→S_0_ decay is enhanced by such a structure. This is due to a reduction in excited-state flattening caused by bulky ligands. Therefore, the molecule is kept closer to a tetrahedral geometry. This geometry brings the d_1_ and d_2_ orbitals closer in energy, enhancing spin–orbit coupling (SOC). Moreover, between complexes **1**, **2**, **4**, and **5**, there’s a noticeable relationship between ligand volume and the decrease of Δ*E*_(S1–T1)_, meaning that it is related to the spatial separation between the HOMO and LUMO. This electronic process may also contribute to the improved performance of **4** and **5**.

### Photophysical Interpretation through Theoretical Results

A theoretical study was conducted to deepen the understanding of the complexes’ photophysical properties. The calculations were performed at the ZORA-D3-PBE0/def2-TZVP­(-f) density functional theory level. First, the optimized geometries for all complexes in the mononuclear structure (Figure S33). The optimized outcome for complex **3** showed sufficient agreement with the experimental XRD results, given that **3** was the only molecule to form mononuclear single crystals. The angle and bond length deviations between experimental and theoretical data were between 0.4 and 4.7% (Table S2).

Time-dependent density functional theory is used to describe the excited states of the series. The experimental and theoretical absorption spectra of **1** were compared (Figure S34). A strong correlation is observed between the calculated absorption and the shoulder observed in the experimental curve at around 300 nm. The analysis of TD-DFT results shows that all electronic transitions within this lower energy band have contributions from d­(Cu) + π­(I) → π*­(PPh_3_) orbitals and little to no contributions from imidazole’s orbitals. Therefore, the states involved in the electronic processes in the molecule have ^1^(M+X)­LCT and ^3^(M+X)­LCT character. The absorption at 260 nm is known to be from π­(PPh_3_) → π*­(PPh_3_) transition.^49^ These observations are extended to the other molecules in the series, given that their electronic configurations and transition energies are similar.

The trend of the fluorescence oscillator strength along the series is closely related to that of *k*_PF_ (Table 2). Since it was established that the S_0_ → S_1_ transition has contributions from copper, iodine, and PPh_3_ electrons, the variation of the alkyl ligands in the imidazole moiety does not affect it directly but instead changes the distortion present around the Cu­(I) center. Complexes **4** and **5**, with the bulkier butyl and benzyl ligands, have *f*_PF_ values of 0.0150 and 0.0219, respectively, while complex **1** is 0.0305.

**Table 2:** Comparison between the Rates of Fluorescence and Phosphorescence and the Calculated Oscillator Strength for S_0_→S_1_ and S_0_→T_1_ Transitions

**complex**	***k***_**PF**_ **/s** ^**–1**^	***f*_PF_**	***k***_**Phos**_ **/s** ^**–1**^	***f*_Phos_**
**1**	3.5 × 10^6^	0.0305	3.8 × 10^3^	6.67 × 10^–5^
**2**	7.7 × 10^6^	0.0331	4.0 × 10^3^	8.28 × 10^–5^
**3**	1.8 × 10^7^	0.0355	5.2 × 10^3^	7.27 × 10^–5^
**4**	2.7 × 10^6^	0.0150	8.7 × 10^3^	7.80 × 10^–5^
**5**	3.7 × 10^6^	0.0219	5.7 × 10^3^	9.36 × 10^–5^

The phosphorescence oscillator strength includes SOC effects and correlates with the experimental *k*_Phos_, with deviations, especially for complexes **4** and **5**, which are the molecules with the most significant deviations from the Boltzmann model, not reaching a steady phosphorescence at 77 K. An analysis of the spin–orbit coupling matrix elements (SOCME) between the triplet and singlet states shows that the most significant values of coupling are achieved between the triplet T_1_ and the singlets S_2_ and S_4_ (Table 3). On top of that, complexes **3**, **4**, and **5** show the highest matrix elements, fitting with the highest *k*_Phos_, induced by efficient SOC due to a restriction of molecular flattening in the excited state. These couplings relate to d­(Cu) → π*­(PPh_3_), and the impact of the different imidazole derivatives lies in the disturbance of the triphenylphosphine’s benzene rings caused by the bulky alkyl chain.

**Table 3:** SOCME Data Calculated in Optimized S_0_ Geometry within ZORA-D3-PBEO/def2-TZVP­(-f) Theory Level^a^

**complex**	**⟨T**_**1**_ **|*H***_**SOC**_ **|S**_**1**_ **⟩** ^b^	**⟨T**_**1**_ **|*H***_**SOC**_ **|S**_**2**_ **⟩** ^b^	**⟨T**_**1**_ **|*H***_**SOC**_ **|S**_**3**_ **⟩** ^b^	**⟨T**_**1**_ **|*H***_**SOC**_ **|S**_**4**_ **⟩** ^b^
**1**	34.89	190.29	84.65	206.75
**2**	40.81	218.11	71.52	177.88
**3**	38.51	196.67	99.08	188.81
**4**	101.64	103.21	51.29	294.72
**5**	51.56	89.81	41.54	323.74

a In cm^–1^.

b $\sqrt{{\sum_{MS}\left\langle T_{j\left(MS=0,\;\pm 1\right)}\right|H_{\mathrm{SOC}}\left|S_{n}\right\rangle }^{2}}$ in S_0_ geometry.

## Conclusions

A series of novel Cu­(I) complexes was synthesized via a cost-effective and time-efficient route. Single-crystal XRD, alongside various structural characterization techniques, confirmed a mononuclear structure of the series. Steady-state measurements revealed identical behavior across the series in solution, whereas slight changes were observed in their powders. Emission via a thermally activated delayed fluorescence (TADF) mechanism was confirmed for complexes **1–5**. Delayed emission persisted at 77 K, enabled by the small Δ*E*_(S1–T1)_ values, which further decreased with longer alkyl chains. The complexes displayed radiative decay rate constants comparable to those of Cu­(I) systems with rigid chelating ligands. However, the increased number of C–C bonds in the flexible ligands introduced nonradiative decay pathways, reducing quantum yields compared to reported [CuX­(PPh_3_)­N]-type materials, especially at room temperature. Notably, the bulkiest ligands led to improved emission at 77 K, attributed to enhanced spin–orbit coupling (SOC) due to the d-orbital proximity in the near-tetrahedral geometry. Theoretical analysis supported the experimental findings, showing that the imidazole moiety does not contribute to the frontier orbitals. Thus, variations in electronic behavior arise solely from ligand structure and spatial arrangement around the metal center. These insights highlight the photophysical performance of blue-emitting Cu­(I) complexes, employing simple monodentate ligands and metals, and demonstrate how emission properties can be tuned through simple ligand modifications.

## Experimental Section

### General Procedures

All reagents were purchased from commercial sources, Sigma-Aldrich, Merck, Acros, and Vetec, and used without further purification. Reactions were monitored by Thin Layer Chromatography (TLC) on aluminum plates coated with a thin layer of silica gel 60, with the indicator UV254 Marcherey – Nagel. The percentages of carbon, nitrogen, and hydrogen of the synthesized complexes were acquired with a PerkinElmer 2400 Series II CHNS/O Elemental Analyzer. Infrared spectra were obtained with a Bruker α spectrometer in the range between 400 and 4000 cm^–1^. The samples were analyzed as KBr pellets. ^1^H, ^13^C, and ^31^P NMR spectra were recorded with an AVANCE DRX spectrometer operating at 200, 50, and 43 MHz, respectively. Chemical shifts (δ) are given in parts per million (ppm) and are relative to those of tetramethylsilane (TMS) as an internal standard. The solvents used to obtain the NMR spectra were deuterated chloroform (CDCl_3_) and deuterated dichloromethane (CD_2_Cl_2_). Thermogravimetric analysis was carried out on a Shimadzu TGA-50 analyzer using about 5 mg of the materials under a nitrogen flow of 50 mL min^–1^. The samples were heated to 900 °C at a rate of 10 °C/min. The redox behavior was studied through cyclic voltammetry, with an Autolab PGSTAT302N potentiostat in the range from −0.5 to 0.8 V. The measurements were carried out on a 2 × 10^–3^ mol L^–1^ solution of the complexes in dichloromethane with NBu_4_PF_6_ as supporting electrolyte. Glassy carbon was used as the work electrode, platinum as the reference electrode, and graphite as the counter electrode. Ferrocene was used to correct the reference electrode. For more in-depth characterization, single-crystal XRD was performed. Single crystals were obtained via slow diffusion using chloroform as the solvent and diethyl ether as the antisolvent. The single-crystal structures were elucidated using a Bruker APEX II DUO diffractometer with a molybdenum tube as the radiation source and a graphite monochromator.

### Optical Spectroscopic Measurements

Electronic absorption spectra of the complexes in chloroform solution (4 × 10^–5^ mol L^–1^) were recorded using the OceanOptics USB4000 spectrometer and the photoluminescence (PL) with a Hitachi F7000 fluorimeter, which was also used for the excitation and PL measurements in powder. The powder absolute photoluminescence quantum yields (PLQY) were obtained with a Hamamatsu Photonics c9920–02G spectrophotometer by using an integrating sphere under 400 nm excitation. Singlet excited-state decay curves were recorded by using the time-correlated single photon counting (TCSPC) method with a Horiba Scientific FL3–22-iHR320 spectrophotometer. Time-resolved emission spectra were obtained by exciting the samples with a Nd:YAG pulsed laser (EKSPLA) at 355 nm and 10 Hz, and the emission was captured with a Stanford Computer Optics gated intensified CCD camera.

### Theoretical Methods

The calculations were performed on the software Orca 5.0.3 at the DFT level.^50^ PBE0 was employed as the exchange-correlation function,^51^ seeing as it provided agreeable accuracy in the description of the Cu­(I) atoms within the tested methods (B3LYP, PBE0, M06, and ωB97X). Geometry optimizations were carried out with the basis set ZORA-def2-TZVP for the copper atom, old-ZORA-TZVP for iodine, and ZORA-def2-TZVP­(-f) for the remaining atoms.^52,53^ The calculations also included D3 dispersion correction along with BJ damping, developed by Grimme.^54,55^ RIJCOSX algorithm^56,57^ was introduced to speed up the resolution of the integrals using the RIJ approximation and the chain-of-spheres approach for exchange (COSX). RIJ requires an auxiliary basis set for the Coulomb part (Def2/J) and a numeric integration grid for the exchange part (DefGrid-2). The possible structures resolved by XRD were utilized initially, and the SCF and geometry convergence criteria were classified as very tight. Time-dependent density functional theory (TD-DFT)^58^ was employed using the equilibrium geometry (S_0_), including only the minimum number of lowest-energy singlet–singlet excitations to reproduce the absorption spectra in the UV–vis region. The same above-mentioned protocol was applied, along with the linear response conductor-like polarizable continuum model (LR-CPCM)^59^ to account for solvent effects. In this approach, chloroform was represented as a continuum polarizable dielectric. SOC matrix elements among the first singlet–triplet states were obtained through the SOC-TD-DFT model.^60,61^ SOC integrals used here were calculated using the RI-SOMF­(1X) mean-field method.^62^ The geometric representations of the molecules were obtained by using Chemcraft software.

## Supplementary Material



## Notes

It includes ^1^H NMR, ^13^C NMR, ^31^P­{^1^H} NMR, and IR spectra; photophysical and theoretical calculations data; X-ray crystallography CCDC: 2442778 (PDF)
