# Supplementary material for: Influence of Imidazole Substituent Bulkiness on [CuI(PPh3)2N] Complexes with TADF Blue Solid-State Emission
Source: ACS Omega. 2026 Feb 19;11(8):13425–34. doi: 10.1021/acsomega.5c10663 (PMC12961488; doi:10.1021/acsomega.5c10663)
Supplement: Supplementary file 1 [file ao5c10663_si_001.pdf]

SUPPLEMENTARY INFORMATION

Influence of imidazole substituent bulkiness on [CuI(PPh$_3$)$_2$N] complexes with TADF blue solid-state emission

Carolina Francener[1,5]*, Giliandro Farias[2], Renê Santos de Amorim[1], Marcelo Meira Faleiros[3], Larissa Gomes Franca[4,5], Andrew P. Monkman[5], Adailton J. Bortoluzzi[1], Teresa Dib Zambon Atvars[3], Eduard Westphal[1], Ivan H. Bechtold[6]*.

[1]*Universidade Federal de Santa Catarina, Department of Chemistry, R. Agronomico Andrei Cristian Ferreira, 88040-900, Florianópolis, Brazil*

[2]*Universidade de São Paulo, Department of Materials Physics and Mechanics, R. Da Reitoria, 05508-220, São Paulo, Brazil*

[3]*Universidade Estadual de Campinas, Department of Chemistry, Cidade Universitaria Zeferino Vaz, 13083-970 Campinas, SP, Brazil*

[4]*University of Cambridge, Department of Materials Science and Metallurgy, Trinity Ln, CB2 1TN Cambridge, UK*

[5]*Durham University, Department of Physics, South Rd, DH1 3LE, Durham, UK*

[6] *Universidade Federal de Santa Catarina, Department of Physics, R. Agronomico Andrei Cristian Ferreira, 88040900, Florianópolis, Brazil*

*Corresponding authors: carolinasfrancener@gmail.com, ivan.bechtold@ufsc.br

1. Synthesis and characterization

All reagents were purchased from commercial sources, Sigma-Aldrich, Merck, Acros, Vetec, and used without further purification. 1-methylimidazole (Me-Im) was obtained from commercial sources. Reactions were monitored by Thin Layer Chromatography (TLC) on aluminum plates coated with a thin layer of silica gel 60, with the indicator UV254 – Marcherey – Nagel.

1.1 Ligands

1-ethylimidazole (Et-Im). Imidazole (29.4 mmol, 2.00 g), ethyl bromide (32.4 mmol, 3.52 g), potassium carbonate (58.8 mmol, 8.12 g), and acetone (20 mL) were added to a round-bottom flask equipped with a condenser. The solution was stirred under reflux overnight. The acetone was removed under reduced pressure, and the remaining solid was dissolved in 20 mL of DCM and 20 mL of water and transferred to a separatory funnel. The organic phase was separated and washed 3 times with 10 mL of 5% NaOH (w/w) solution and once with water. The organic phase was separated, dried over anhydrous sodium sulphate, and the solvent was rotary evaporated and used for complexation without further purification. The product was obtained as an oil, with a 64% yield. The NMR data are in accordance with those

reported[1]. **^{1}H RMN** ($CDCl_3$, 200 MHz) δ (ppm): 1.43 (t, *J* = 7.3 Hz, 3H, CH_3); 3.98 (q, *J* = 7.3 Hz, 2H, CH_2); 6.92 (s, 1H, R-N-CH-CH-N); 7.03 (s, 1H, R-N-CH-CH-N); 7.47 (s, 1H, R-N-CH-N).

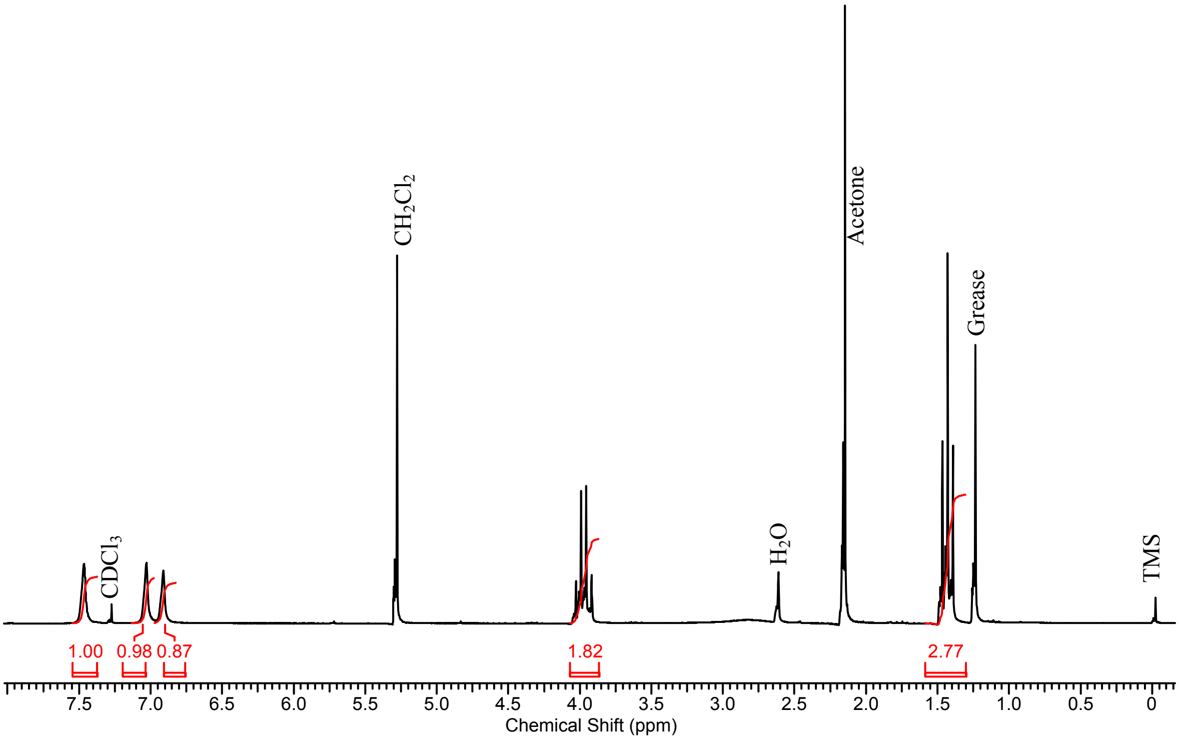

Figure S1 - ^{1}H NMR 200 MHz spectra of Et-Im in $CDCl_3$.

1-isopropylimidazole (Iso-Im). Iso-Im was synthesised in the same way as 1-ethylimidazole, except that ethyl bromide was substituted with isopropyl bromide (89 mmol, 11 g). The product was obtained as an oil with a 71% yield. The product was used for complexation without further purification. The NMR data are in accordance with those reported[2]. **^{1}H RMN** ($CDCl_3$, 200 MHz) δ (ppm): 1.8 (d, J = 6.7, 6H, CH_3); 4.34 (sept, J = 6.7, 1H, CH_3-<u>CH</u>-CH_3); 6.96 (s, 1H, R-N-<u>CH</u>-CH-N); 7.05 (s, 1H, R-N-CH-<u>CH</u>-N); 7.53 (s, 1H, R-N-<u>CH</u>-N).

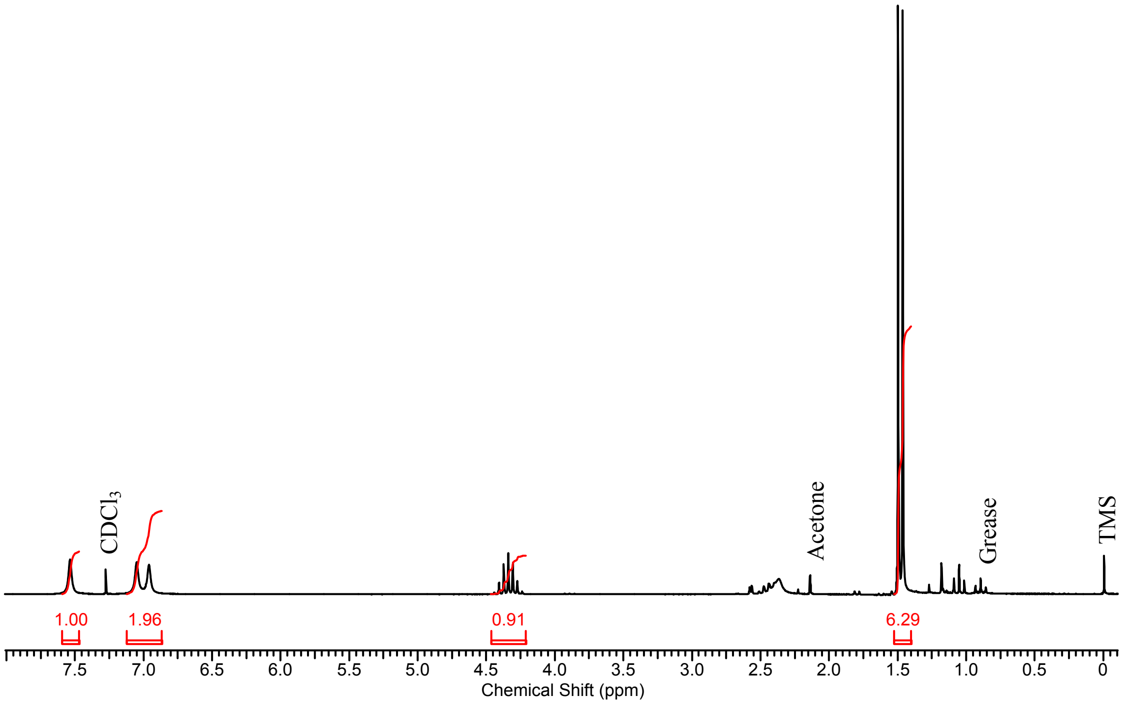

Figure S2 - ^{1}H NMR 200 MHz spectra of Iso-Im in $CDCl_3$.

1-Butylimidazole (But-Im). But-Im was synthesised in the same way as 1-ethylimidazole, except that ethyl bromide was substituted with butyl bromide (32.4 mmol, 4.44 g). The product was obtained as an oil with a 43% yield. The NMR data are in accordance with those reported[2]. The product was used for complexation without further purification **^{1}H RMN** ($CDCl_3$, 200 MHz) δ (ppm): 0.94 (t, J = 7.2 Hz, 3H, CH_3); 1.30 (m, 2H, N-CH_2-CH_2-CH2-CH_3); 1.75 (m, 2H, N-CH_2-CH_2-CH2-CH_3); 3.92 (t, J=7.2 Hz, 2H, N-CH_2-CH_2-CH2-CH_3); 6.90 (s, 1H, R-N-CH-CH-N); 7.04 (s, 1H, R-N-CH-CH-N); 7.45 (s, 1H, R-N-CH-N).

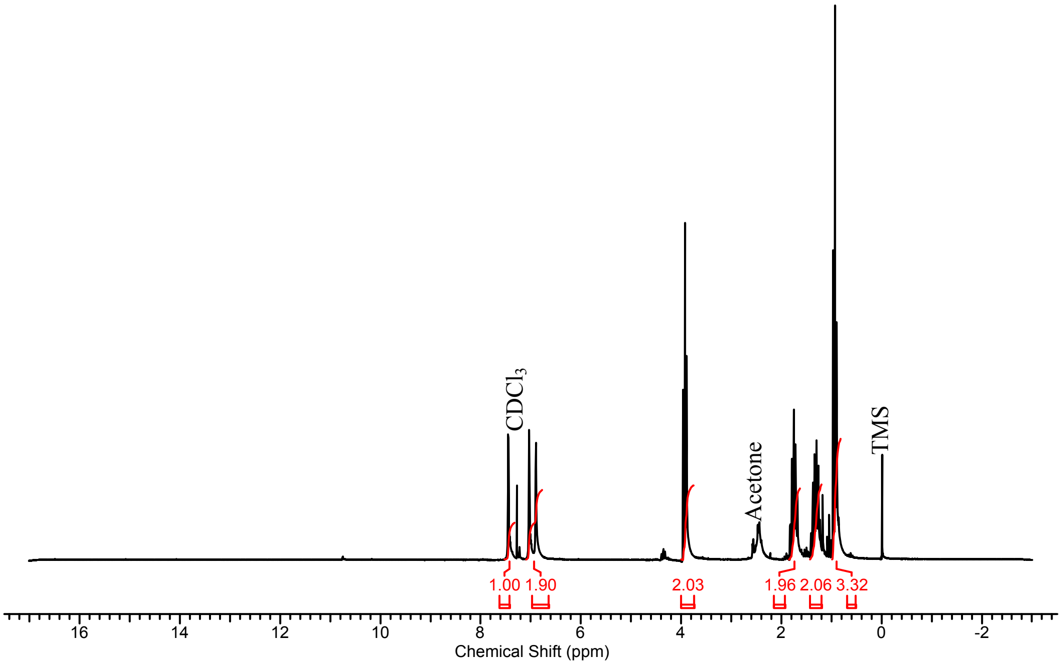

Figure S3 - ^{1}H NMR 200 MHz spectra of But-Im in $CDCl_3$.

1-Benzylimidazole (Ben-Im). Ben-Im was synthesised in the same way as 1-ethylimidazole, except that ethyl bromide was substituted with benzyl bromide (32.4 mmol, 4.44 g). The crude oil obtained after the rotary evaporation was purified by recrystallization in heptane. The product was obtained as needle-shaped crystals. The NMR data are in accordance with those reported[3]. The product was used for complexation without further purification **^{1}H RMN** ($CDCl_3$, 200 MHz) δ (ppm): 5.13 (s, 2H, CH_2); 6.92 (s, 1H, R-N-<u>CH</u>-CH-N); 7.00 – 7.45 (m, 6H, H-ϕ e R-N-CH-<u>CH</u>-N); 7.56 (s, 1H, N-<u>CH</u>-N).

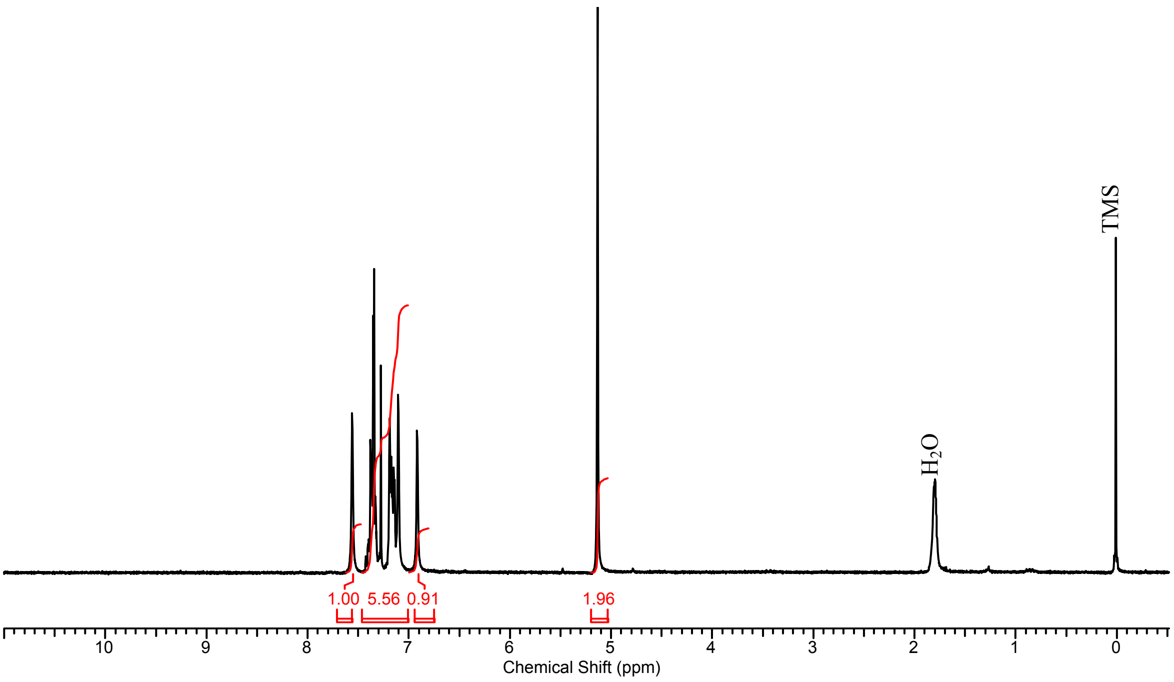

Figure S4 - ^{1}H NMR 200 MHz spectra of Ben-Im in $CDCl_3$.

1.2 Complexes

CuI(PPh₃)(Me-Im)(**1**). Copper iodide (CuI) (0.25 mmol, 0.047 g) and triphenylphosphine (PPh_3) (0.5 mmol, 0.131 g) were added to a round-bottom flask containing chloroform (25 mL). The solution was stirred at room temperature for 30 minutes. Then, 1-methylimidazole (Me-Im) (0.25 mmol, 0.020 g) was added, and the stirring continued for 6 hours. The solvent was partially removed, and the resulting mixture was then poured into ethyl ether to precipitate the product. The white, crystalline-looking solid was filtered under vacuum, yielding 0.15 g (74%) of the pure product. ^1H NMR (200 MHz, CD_2Cl_2) δ ppm: 3.67 (s, 3H) ; 6.90 (s, 2H) ; 7.38 (m, 30H) ; 7.61 (s, 1H). ^{13}C RMN (50 MHz, $CDCl_3$) δ ppm: 33.55 ; 120.09 ; 128.40 (d, J = 8.6 Hz) ; 129.40 ; 133.94 (d, J = 15.3 Hz) ; 139.23. ^{31}P RMN (43 MHz, $CDCl_3$) δ ppm: -4.73. $C_{40}H_{36}CuIN_2P_2$ (797.13 g.mol^{-1}) Calculated C: 60.27% H: 4.55% N: 3.51% Experimental C: 59.85% H: 4.57% N: 3.56%

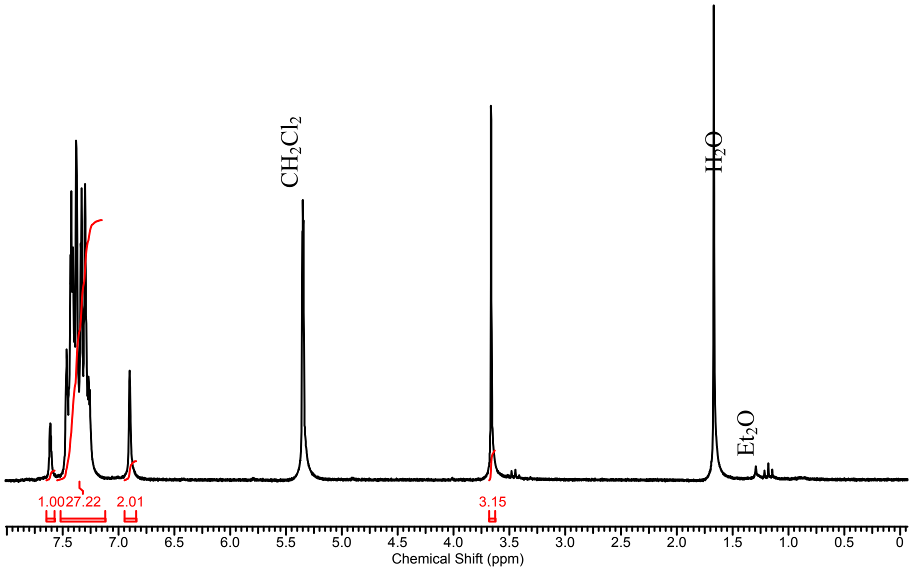

Figure S5 - ^1H NMR 200 MHz spectra of **1** in CD_2Cl_2.

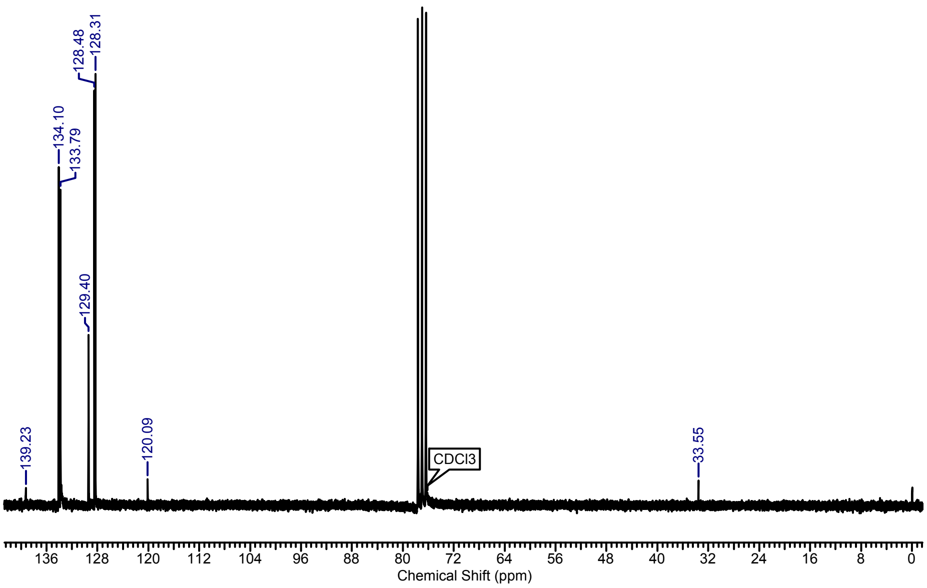

Figure S6 - ^{13}C NMR 50 MHz spectra of **1** in $CDCl_3$.

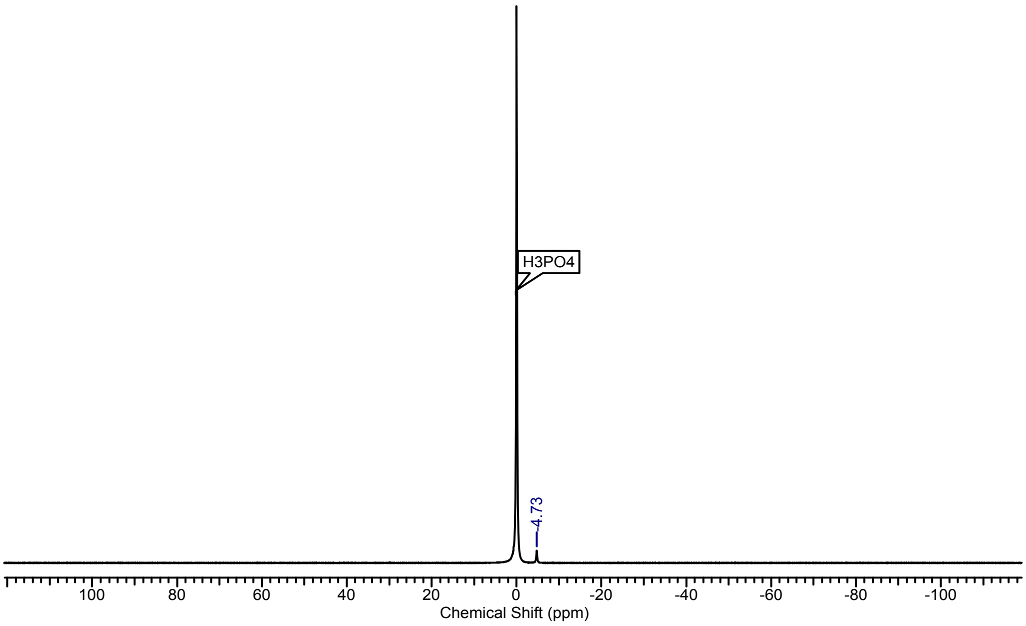

Figure S7 - $^{31}P\{^1H\}$ NMR 43 MHz spectra of **1** in $CDCl_3$.

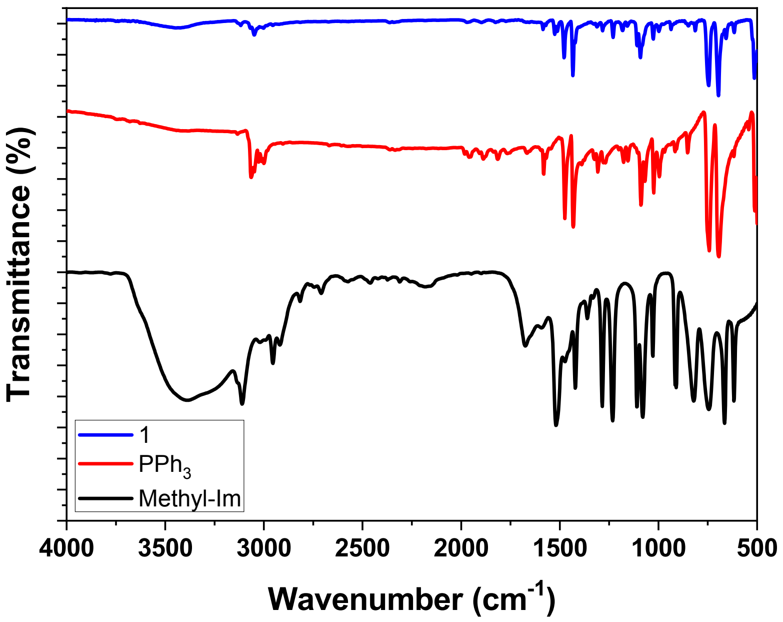

Figure S8 - IR spectra of **1** and respective ligands.

CuI(PPh$_3$)(Et-Im)(**2**). **2** was synthesized the same way as **1**, except that (Me-Im) was replaced with 1-ethylimidazole (Et-Im)(0.25 mmol, 0.024 g). 0.16 g of the product was obtained, with a 79% yield. ^{1}H NMR (200 MHz, CD_2Cl_2) δ ppm: 1.41 (t, 3H, J = 7.3 Hz) ; 3.97 (quad, 2H, J = 7.3 Hz) ; 6.91 (s, 1H) ; 6.94 (s, 1H), 7.37 (m, 30H) ; 7.67 (s, 1H). ^{13}C RMN (50 MHz, $CDCl_3$) δ ppm: 16.09 ; 42.06 ; 118.44 ; 128.42 (d, J = 2.9 Hz); 129.33 ; 129.47 ; 133.31 ; 133.95 (d, J = 15.3 Hz) ; 138.06. ^{31}P RMN (43 MHz, $CDCl_3$) δ ppm: -4.38. $C_{41}H_{38}CuIN_2P_2$ (811.15 g.mol^{-1}) Calculated C: 60.71% H: 4.72% N: 3.45% Experimental C: 60.51% H: 4.56% N: 3.46%

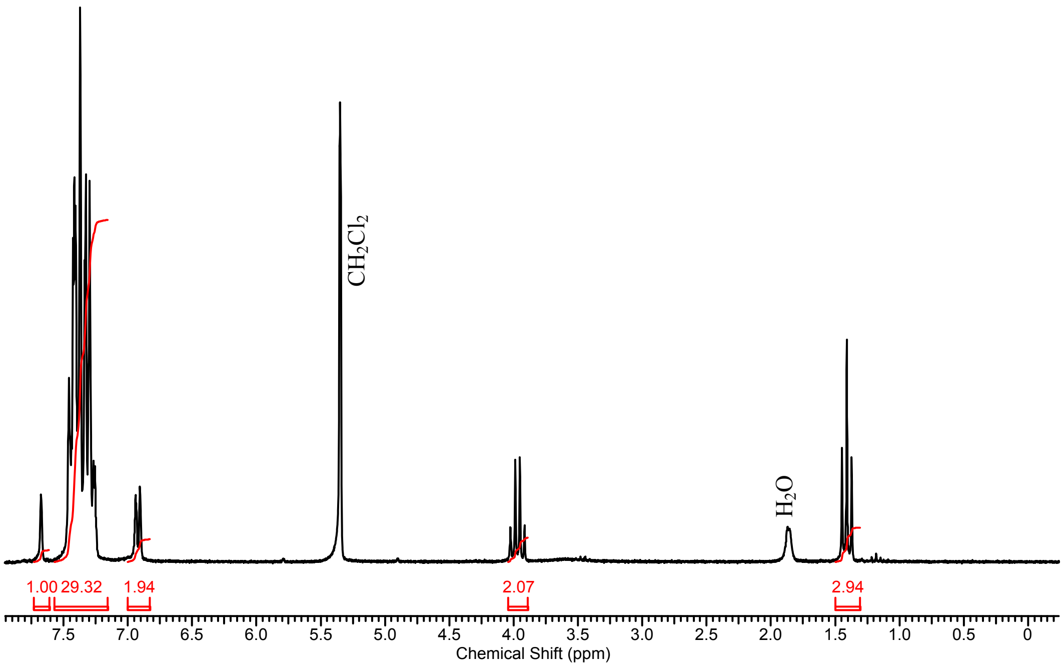

Figure S9 - ^{1}H NMR 200 MHz spectra of **2** in CD_2Cl_2.

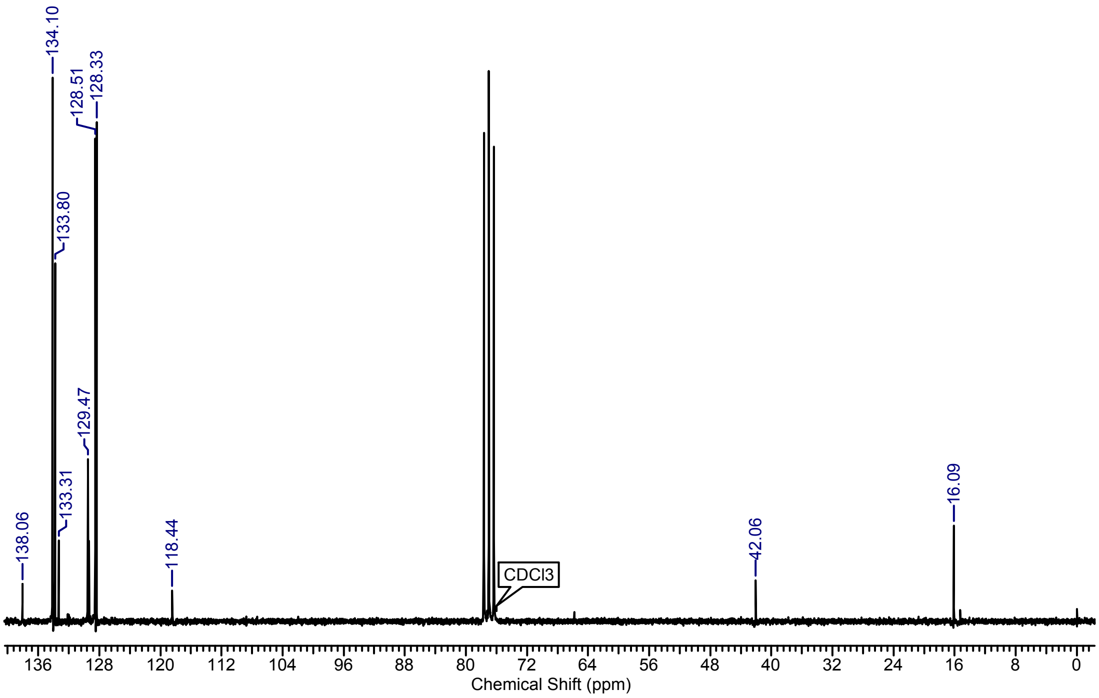

Figure S10 - ^{13}C NMR 50 MHz spectra of **2** in $CDCl_3$.

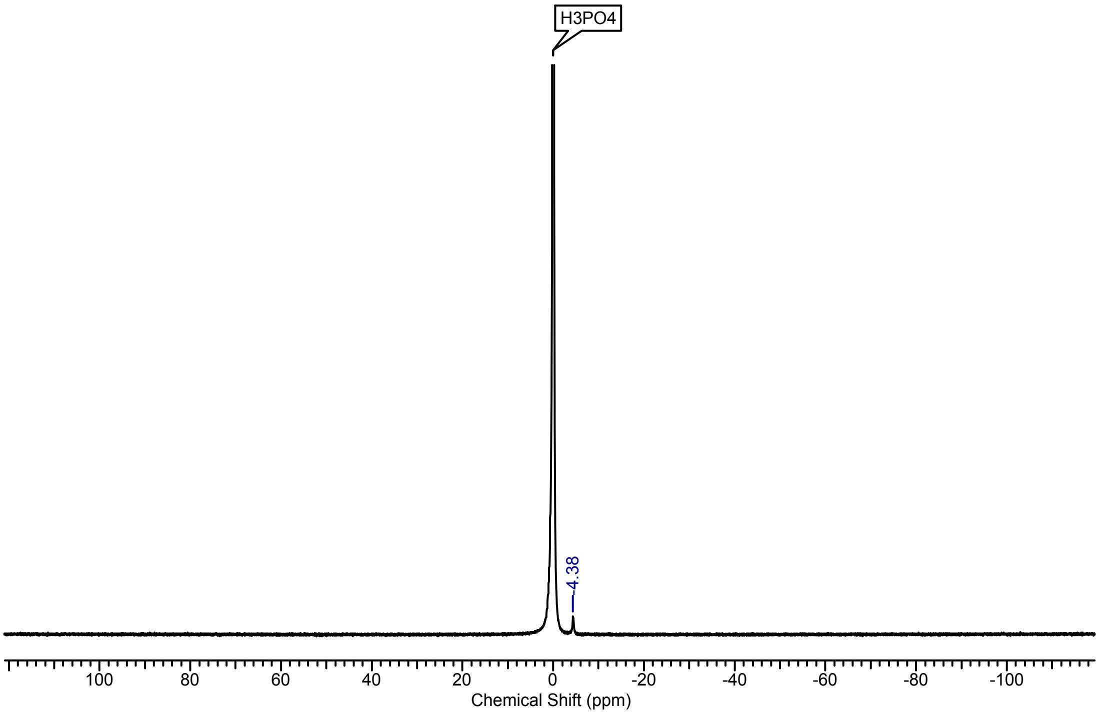

Figure S11 - $^{31}P\{^{1}H\}$ NMR 43 MHz spectra of **2** in $CDCl_3$.

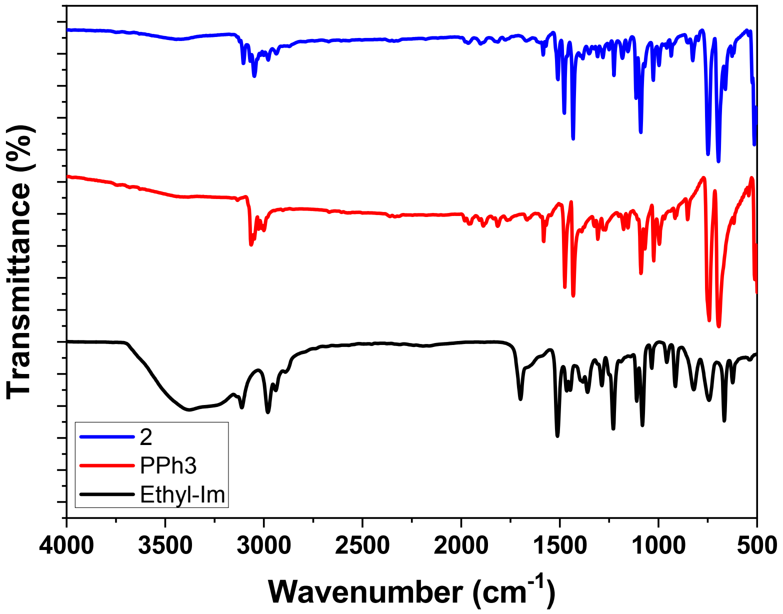

Figure S12 - IR spectra of **2** and respective ligands.

CuI(PPh$_3$)(Pro-Im)(**3**). **3** was synthesized the same way as **1**, except that (Me-Im) was replaced with 1-isopropylimidazole (Pro-Im) (0.25 mmol, 0.027 g). 0.15 g of the product was obtained, with a 73% yield. ^{1}H NMR (200 MHz, CD_2Cl_2) δ ppm: 1.41 (d, 6H, *J* = 6.7 Hz) ; 4.30 (sept, 1H, *J*= 6.7 Hz) ; 6.88 (s, 1H) ; 6.95 (s, 1H) ; 7.32 (m, 30H) ; 7.69 (s, 1H). ^{13}C NMR (50 MHz, $CDCl_3$) δ ppm: 22.53 ; 49.51 ; 116.65 ; 128.38 (d, *J* = 9.0 Hz) ; 129.24 ; 129.43 ; 133.64 (d, *J* = 24.7) ; 133.97 (d, *J* = 14.7 Hz) ; 137.00. ^{31}P NMR (43 MHz, $CDCl_3$) δ ppm: -4.67. $C_{42}H_{40}CuIN_2P_2$ (825.18 g.mol^{-1}) Calculated C: 61.13% H: 4.89% N: 3.39% Experimental C: 60.85% H: 4.91% N: 3.36%

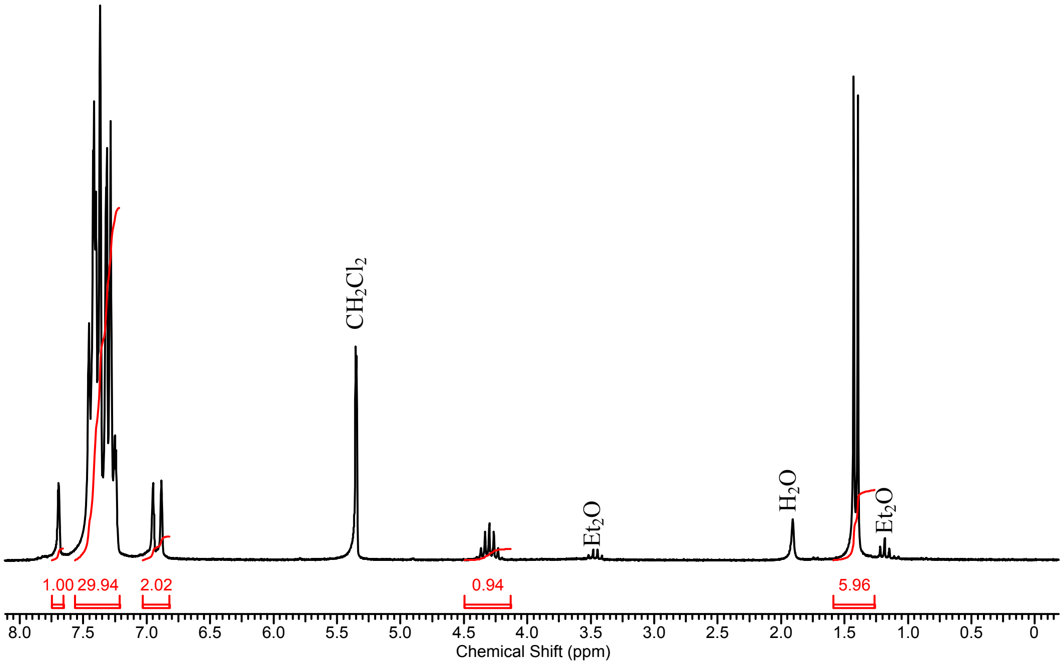

Figure S13 - ^{1}H NMR 200 MHz spectra of **3** in CD_2Cl_2.

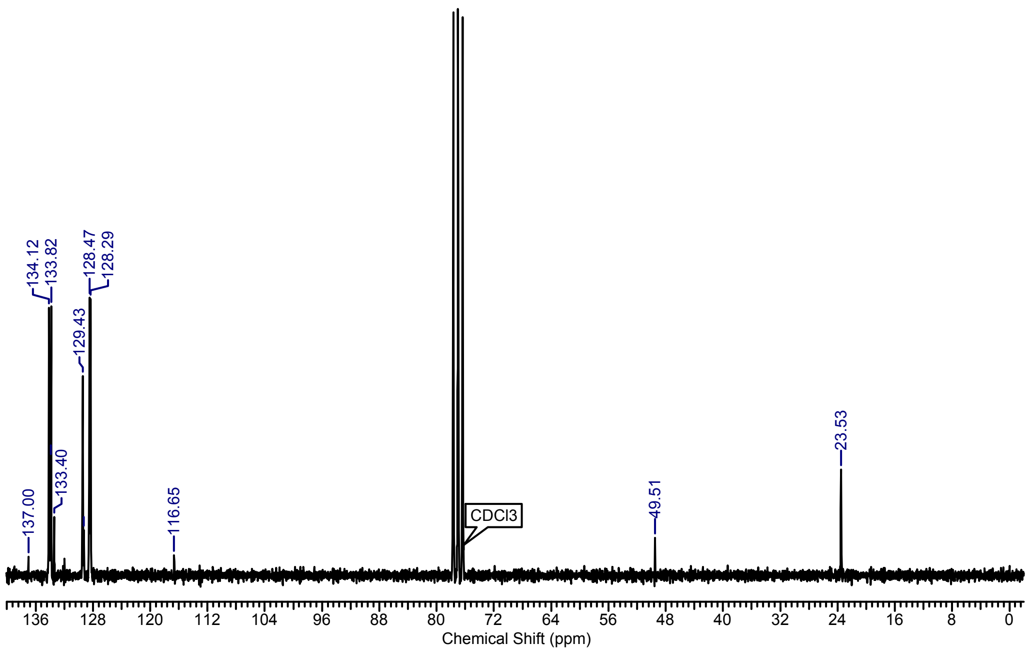

Figure S14 - ^{13}C NMR 50 MHz spectra of **3** in $CDCl_3$.

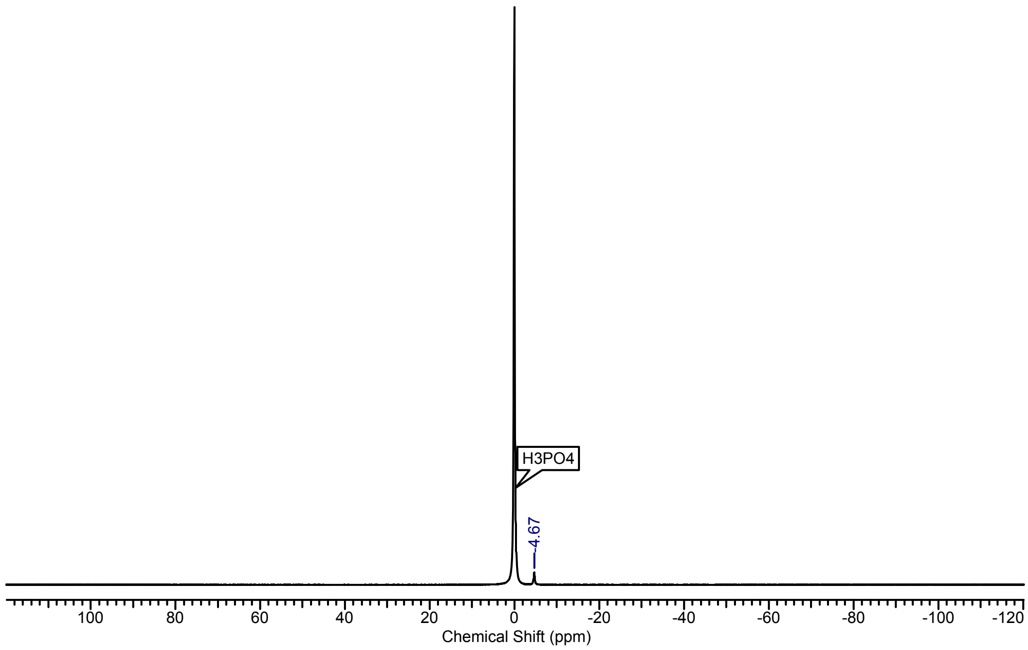

Figure S15 - ^{31}P{^{1}H} NMR 43 MHz spectra of **3** in $CDCl_3$.

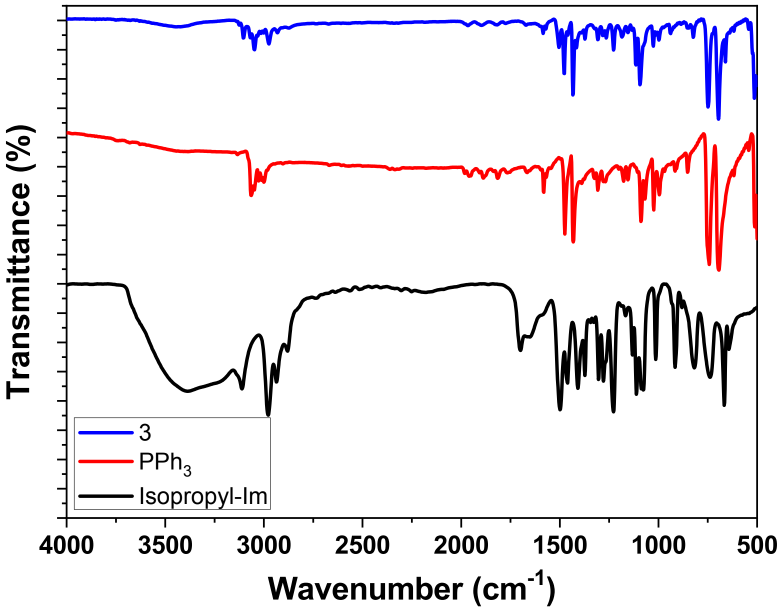

Figure S16 - IR spectra of **3** and respective ligands.

CuI(PPh$_3$)(But-Im)(**4**). **4** was synthesized the same way as **1**, except that (Me-Im) was replaced with 1-butylimidazole (But-Im) (0.25 mmol, 0.031 g). 0.14 g of the product was obtained, with a 67% yield. ^{1}H NMR (200 MHz, CD_2Cl_2) δ ppm: 0.94 (m, 3H) ; 1.26 (m, 2H) ; 1.73 (m, 2H) ; 3.92 (t, *J* = 7.1 Hz, 2H) ; 6.91 (s, 2H) ; 7.37 (m, 30H) ; 7.65 (s, 1H).). ^{13}C NMR (50 MHz, $CDCl_3$) δ ppm: 13.44 ; 19.58 ; 32.08 ; 47.07 ; 118.87 ; 128.37 (d, *J* = 9.0 Hz) ; 129.07 ; 129.40 (d, *J* = 1.3 Hz) ; 133.67 (d, *J* = 24.1 Hz) ; 133.94 (d, *J* = 14.7 Hz) ; 138.52. ^{31}P NMR (43 MHz, $CDCl_3$) δ ppm: -4.72. $C_{43}H_{42}CuIN_2P_2$ (839.20 g.mol^{-1}) Calculated C: 61.54% H: 5.05% N: 3.34% Experimental C: 61.20% H: 5.37% N: 3.08%

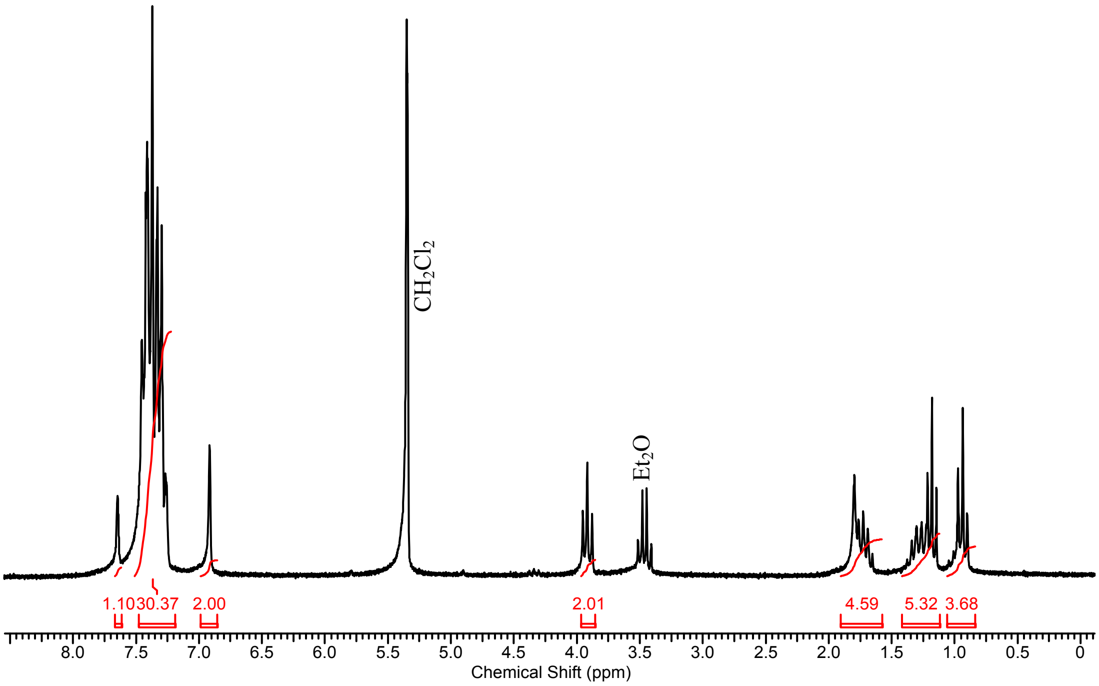

Figure S17 - ^{1}H NMR 200 MHz spectra of **4** in CD_2Cl_2.

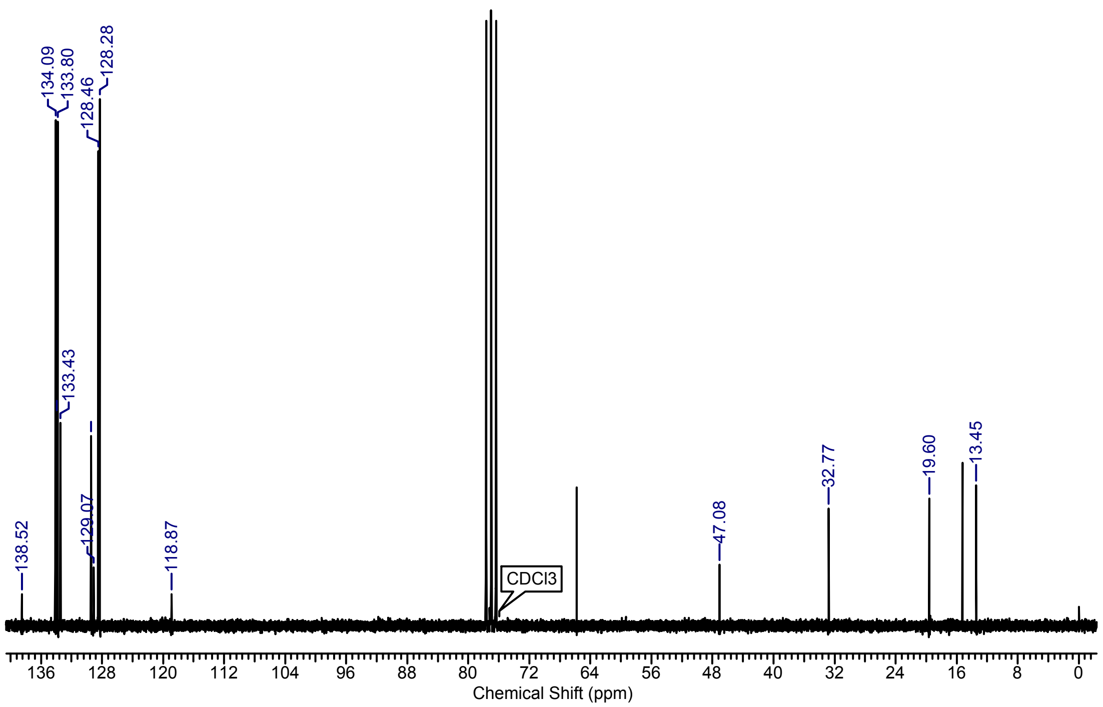

Figure S18 - ^{13}C NMR 50 MHz spectra of **4** in $CDCl_3$.

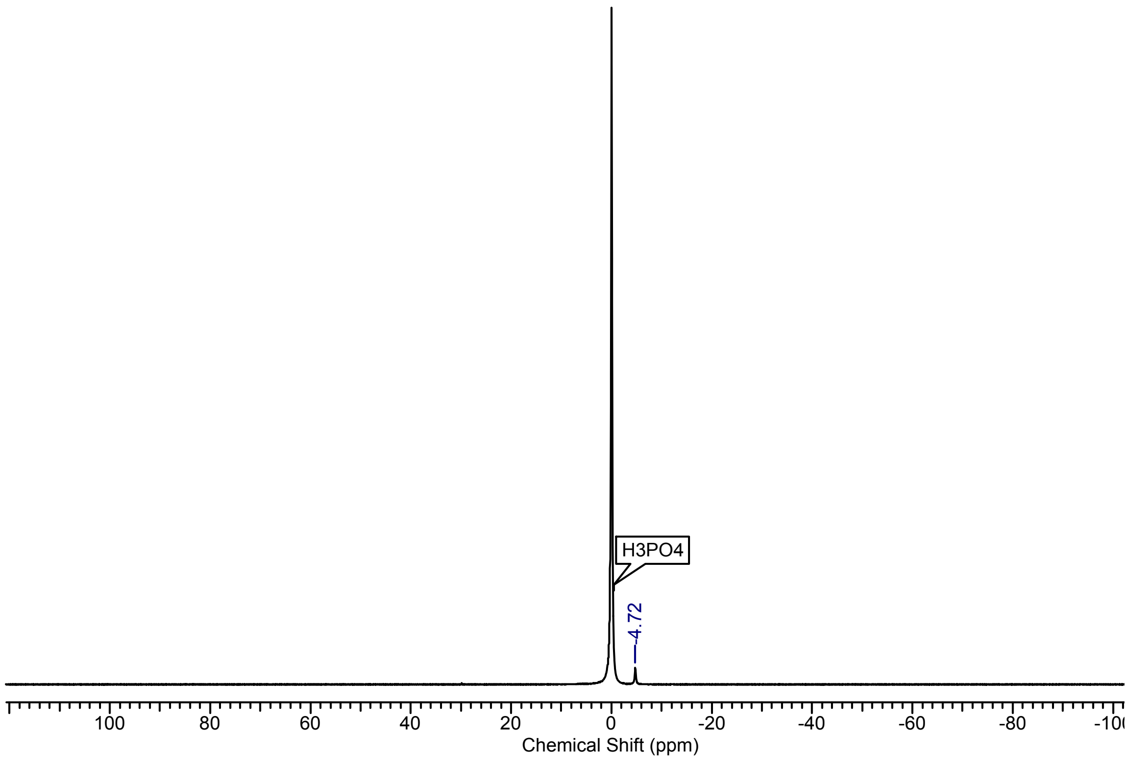

Figure S19 - ^{31}P{^{1}H} NMR 43 MHz spectra of **4** in $CDCl_3$.

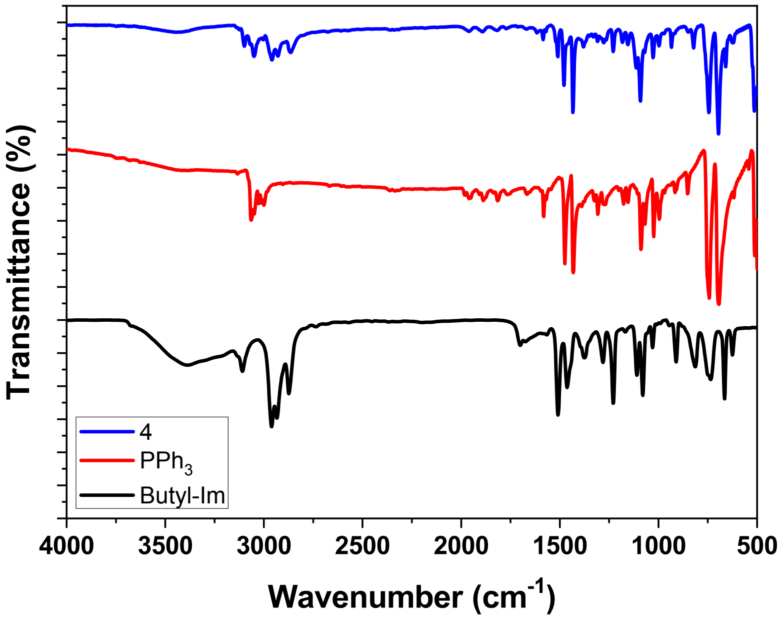

Figure S20 - IR spectra of **4** and respective ligands.

CuI(PPh$_3$)(Bnz-Im)(H$_2$O)$_2$(**5**). **5** was synthesized the same way as **1**, except that (Me-Im) was replaced with 1-benzylimidazole (Bnz-Im) (0.25 mmol, 0.039 g). 0.14 g of the product was obtained, with a 62% yield. ^{1}H NMR (200 MHz, CD_2Cl_2) δ ppm: 5.11 (s, 2H) ; 6.92 (s, 1H) ; 6.97 (s, 1H) ; 7.37 (m , 35H) ; 7.72 (s, 1H). ^{13}C NMR (50 MHz, $CDCl_3$) δ ppm: 51.04 ; 119.26 ; 127.31 ; 128.34 ; 128.38 (d, J = 9.0 Hz) ; 128.99 ; 129.45 (d, J = 1.5 Hz) ; 129.78 ; 133.48 (d, J = 24.9 Hz) ; 133.95 (d, J = 14.9 Hz) ; 135.62 ; 138.87. ^{31}P NMR (43 MHz, $CDCl_3$) δ ppm: -4.83. $C_{46}H_{44}CuIN_2O_2P_2.2H_2O$ (909.25 g.mol^{-1}) Calculated C: 60.76% H: 4.88% N: 3.08% Experimental C: 60.94% H: 4.85% N: 3.13%

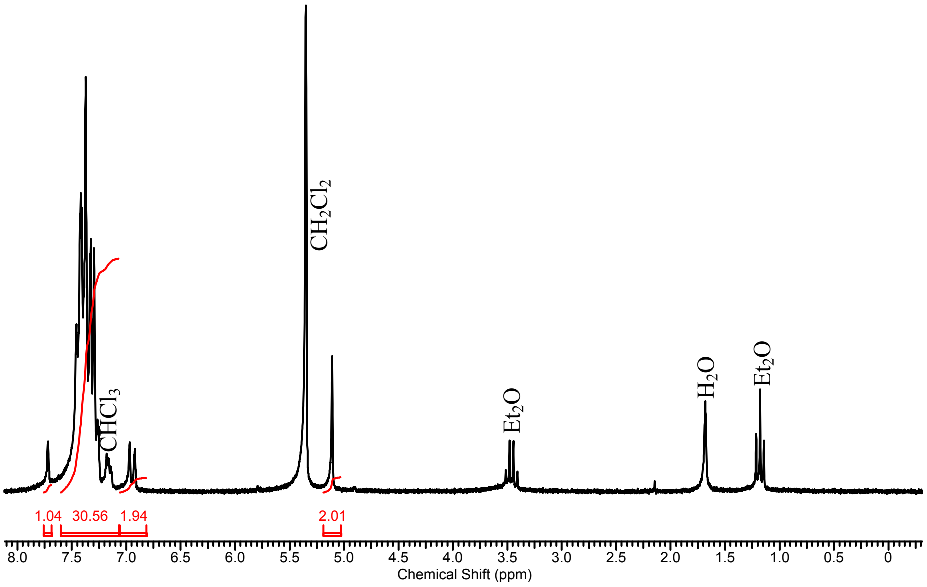

Figure S21 - ^{1}H NMR 200 MHz spectra of **5** in CD_2Cl_2.

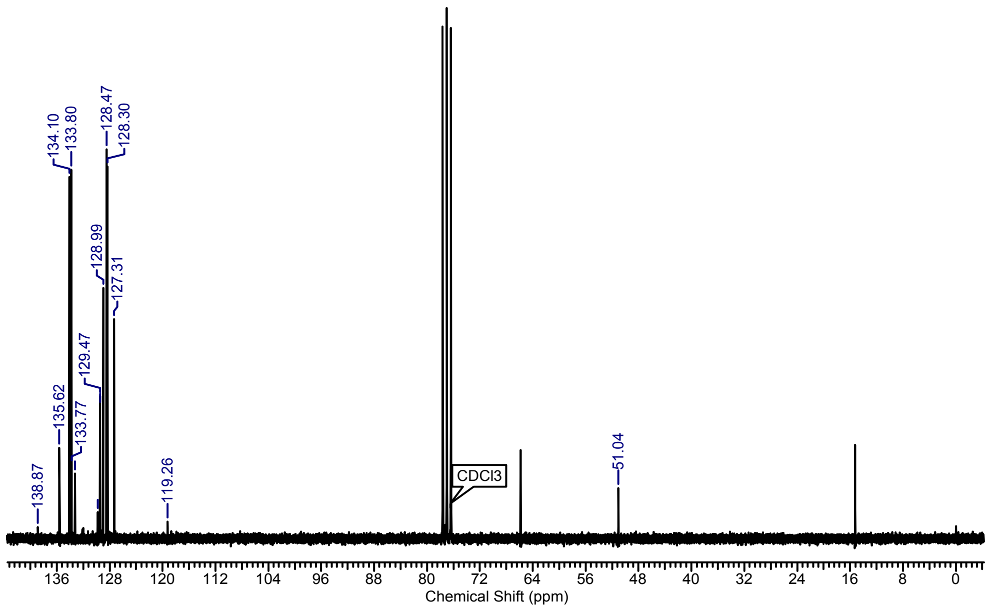

Figure S22 - ^{13}C NMR 50 MHz spectra of **5** in $CDCl_3$.

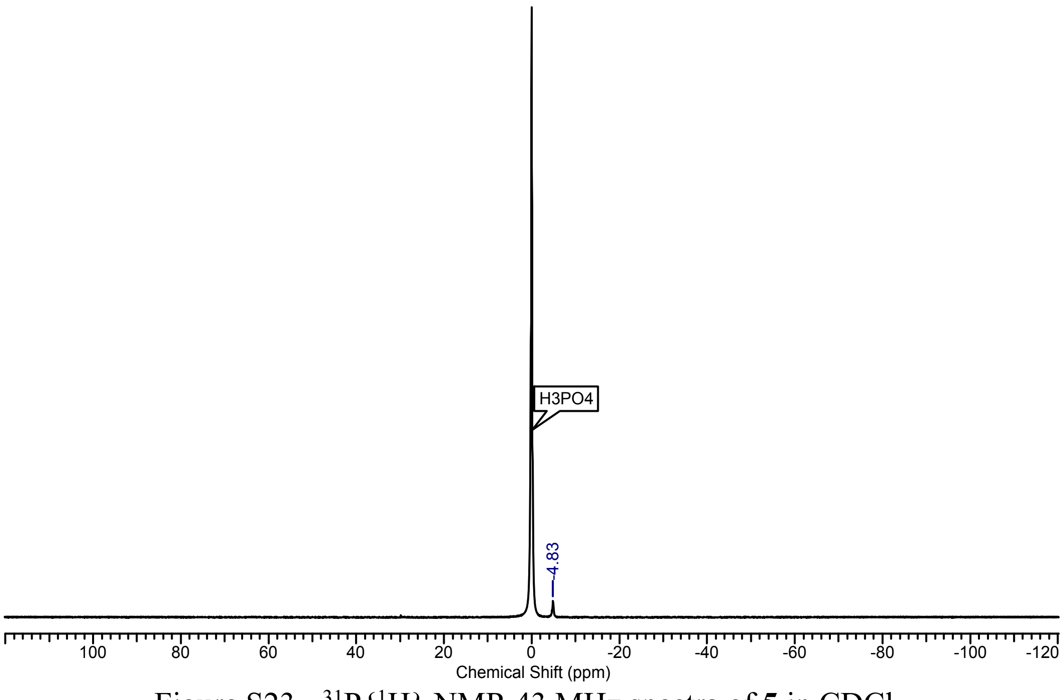

Figure S23 - $^{31}P\{^1H\}$ NMR 43 MHz spectra of **5** in $CDCl_3$.

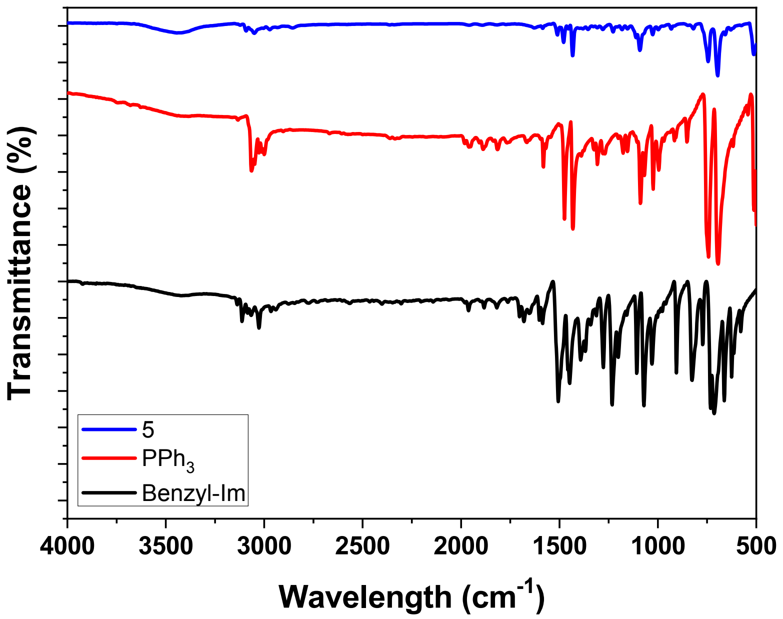

Figure S24 - IR spectra of **5** and respective ligands.

2. Structural characterization

3. Table S1 - Selected bond lengths (A) and angles for **3.**

**Cu(1) – N(1)**	2.042	**N(1)-Cu(1)-P(2)**	114.51
**Cu(1) – P(1)**	2.265	**N(1)-Cu(1)-I(1)**	98.18
**Cu(1) – P(2)**	2.280	**P(1)-Cu(1)-I(1)**	100.75
**Cu(1) – I(1)**	2.706	**P(2)-Cu(1)-I(1)**	107.08
**N(1)-Cu(1)-P(1)**	105.25	**P(2)-Cu(1)-P(1)**	126.54

4. Photophysical characterization

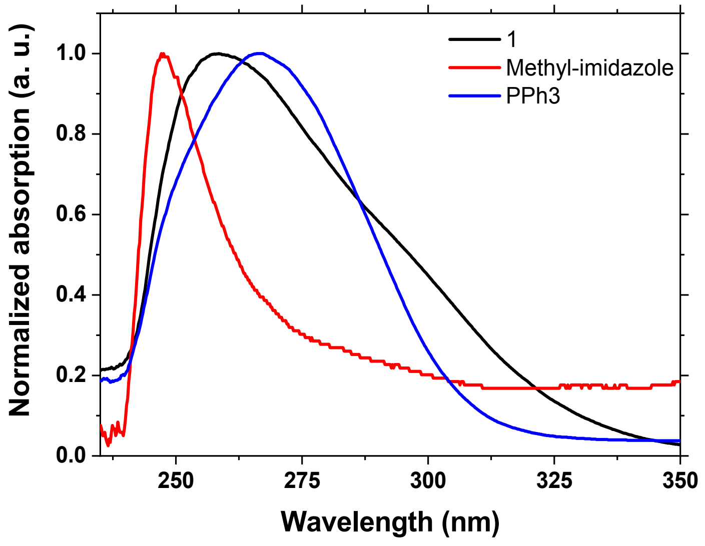

Figure S25 - Absorption spectra of complex 1, methyl-imidazole, and triphenylphosphine (PPh_3) in $CHCl_3$

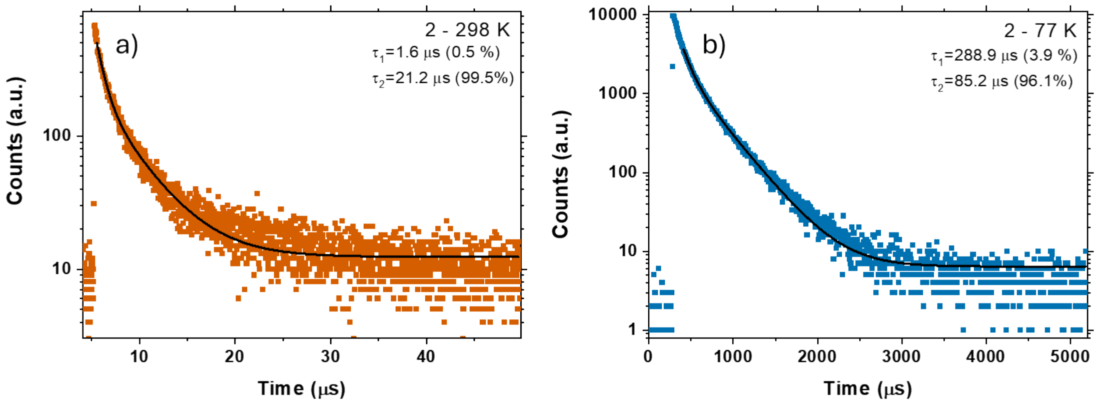

Figure S26 - **a)** Decay curve of 2 in powder form monitored at 450 nm at RT. **B)** Decay curve of 2 in powder form monitored at 450 nm at 77 K.

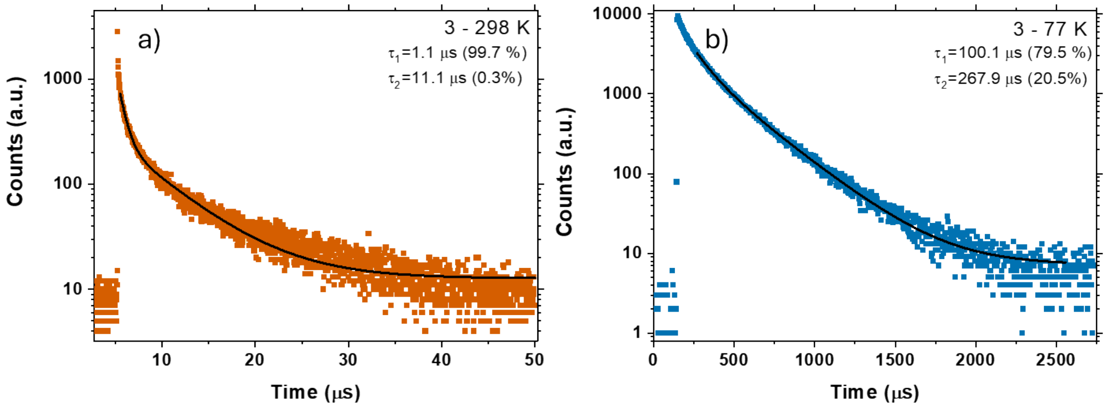

Figure S27 - **a)** Decay curve of 3 in powder form monitored at 450 nm at RT. **B)** Decay curve of 3 in powder form monitored at 450 nm at 77 K.

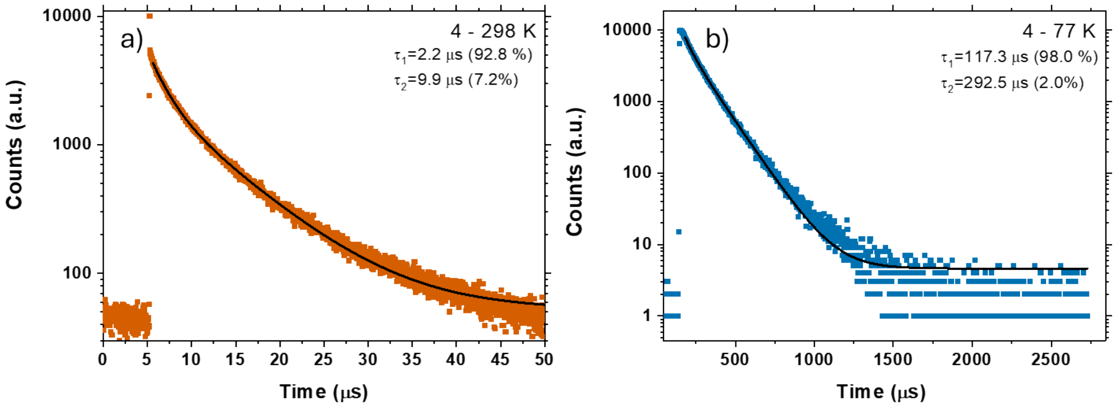

Figure S28 - **a)** Decay curve of 4 in powder form monitored at 450 nm at RT. **B)** Decay curve of 4 in powder form monitored at 450 nm at 77 K.

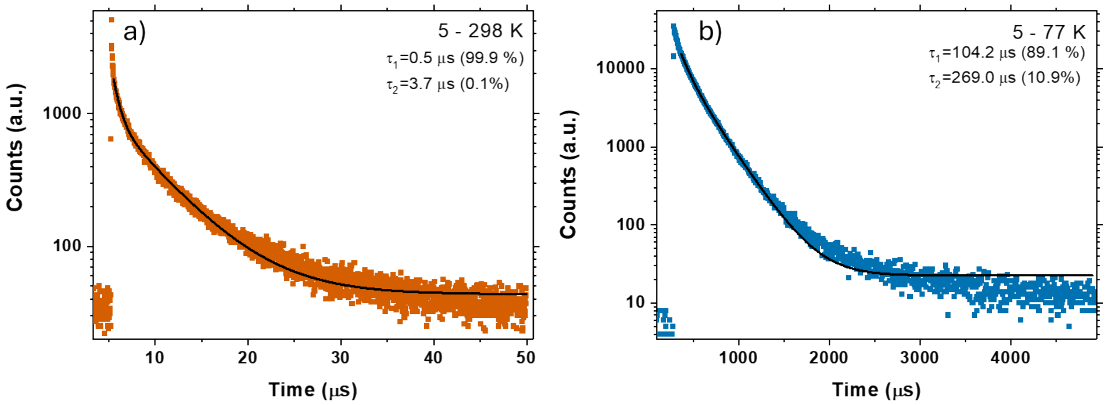

Figure S29 - **a)** Decay curve of 5 in powder form monitored at 450 nm at RT. **B)** Decay curve of 5 in powder form monitored at 450 nm at 77 K.

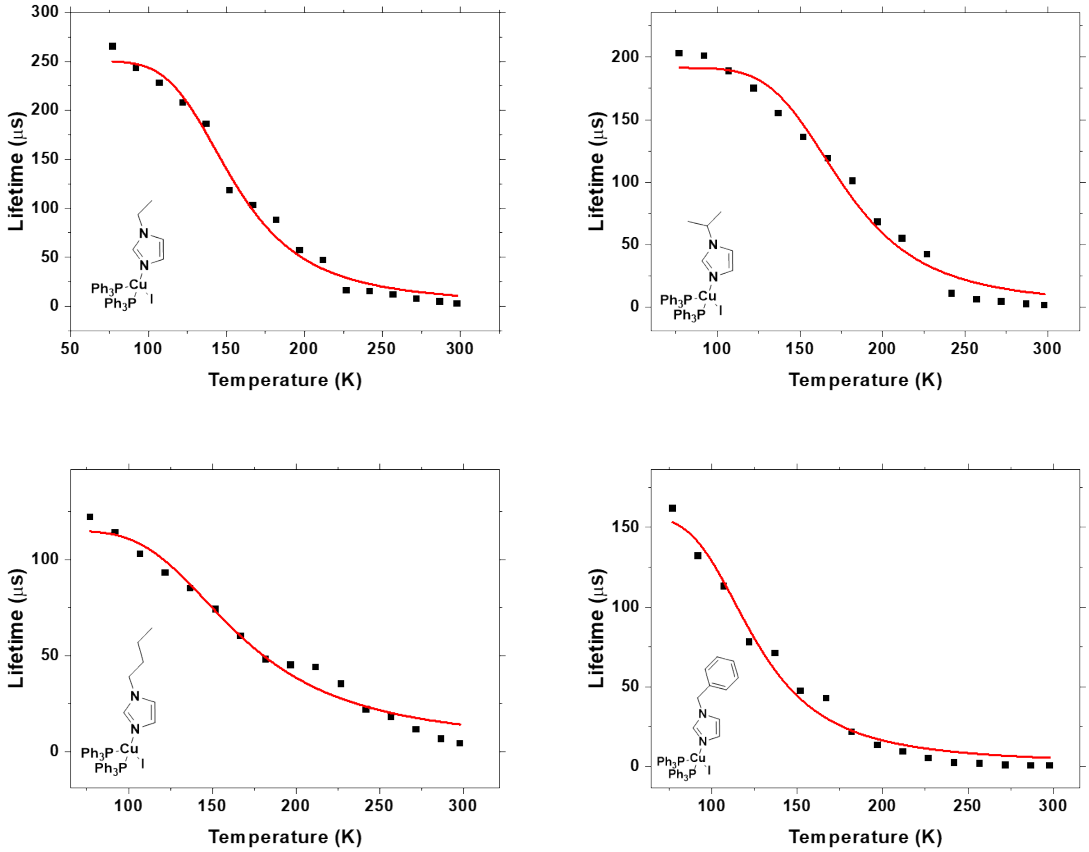

Figure S30 - Temperature-dependent emission lifetime and fitting to the Boltzmann model (Eq S3).

$$\tau(T) = \frac{3 + exp\left(-\frac{\Delta E_{(S1-T1)}}{K_B T}\right)}{3k(T_1) + k(S_1)\, exp\left(-\frac{\Delta E_{(S1-T1)}}{K_B T}\right)} \tag{S1}$$

$$\frac{I(T_1)}{I_{tot}} = \left[1 + \frac{\phi_{PL}(S_1)\tau(T_1)g(S_1)}{\phi_{PL}(T_1)\tau(S_1)g(T_1)}\right] \exp\left(-\frac{E_{(S1-T1)}}{K_B T}\right) \tag{S2}$$

$$\frac{I(S_1)}{I_{tot}} = 1 - \left[1 + \frac{\phi_{PL}(S_1)\tau(T_1)g(S_1)}{\phi_{PL}(T_1)\tau(S_1)g(T_1)}\right] \exp\left(-\frac{E_{(S1-T1)}}{K_B T}\right) \tag{S3}$$

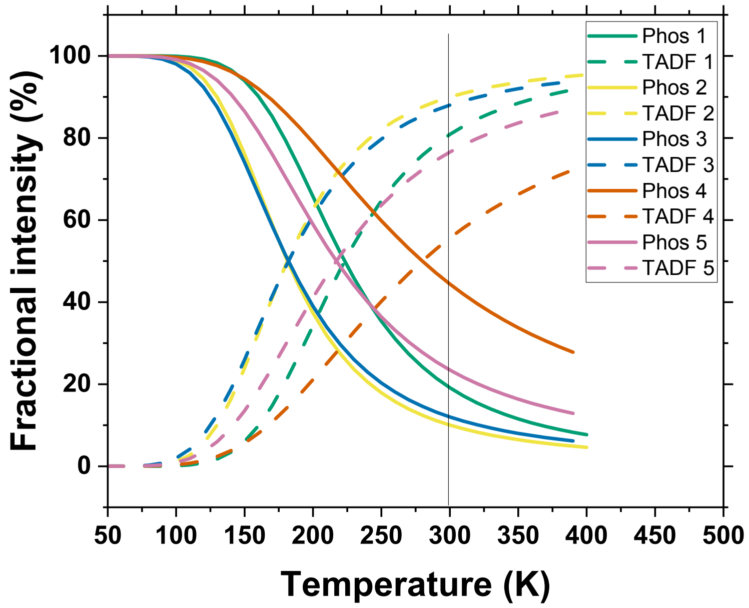

Figure S31 - Temperature dependence of fractional intensity of phosphorescence (dashed) and TADF (solid) in solid state for compounds **1-5**, simulated using eq. (4) and (5); the vertical line indicates room temperature (298 K).

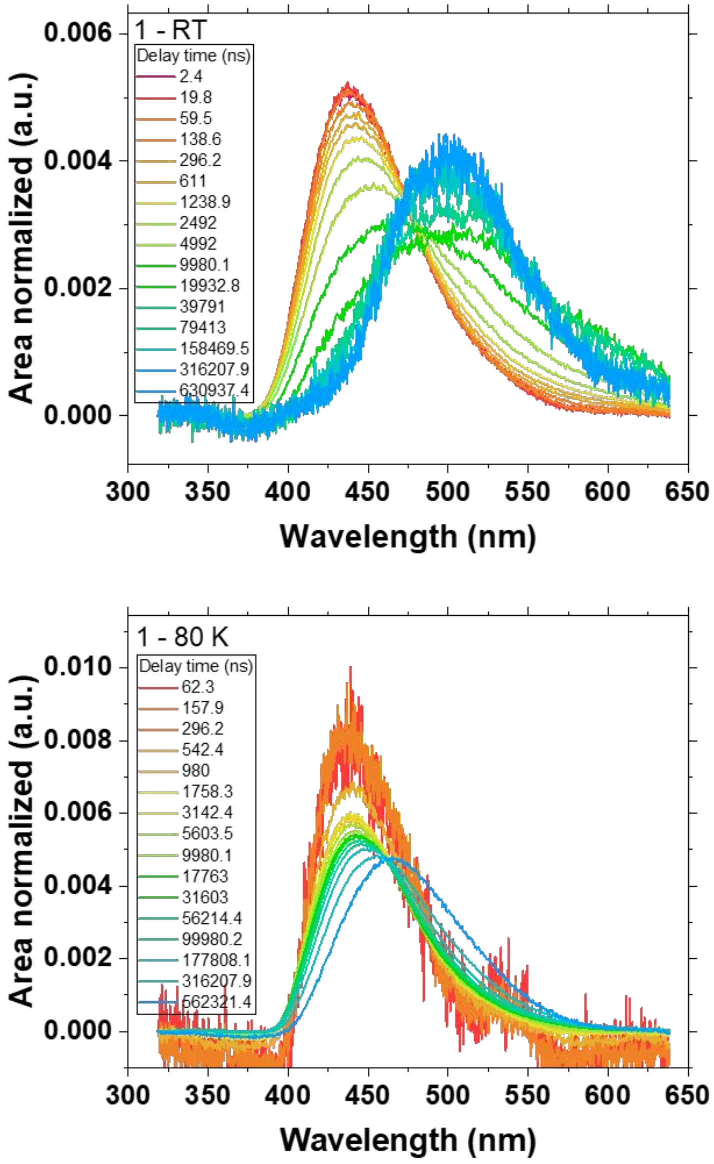

Figure S32 - Time-resolved spectra at room temperature for powder samples under 355 nm excitation.

5. Theoretical calculations

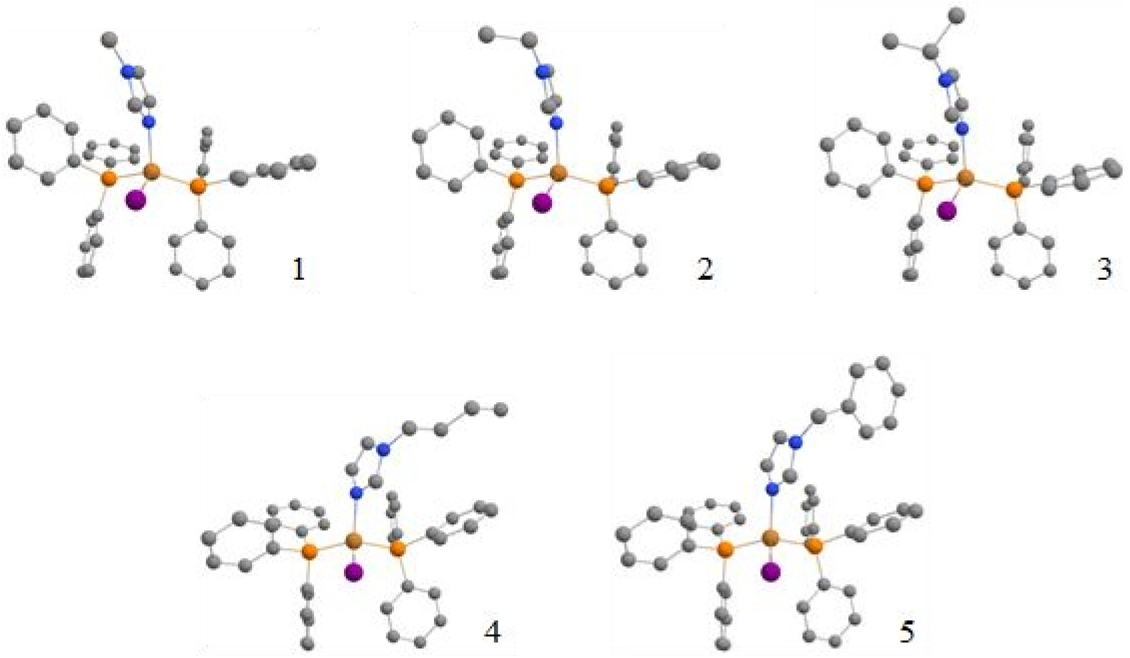

Figure S33 - Optimized ground state geometries for complexes **1-5** within ZORA-D3-PBE0/def2-TZVP(-f).

Table S2 - Bond length and angle calculated within the ZORA-D3-PBE0/def2-TZVP(-f) theory level, along with experimental values and tetrahedral distortion parameter (τ4).

Complexes	1	2	3		4	5
			**Calculated**	**Experimental**		
Cu1-N1	2.083	2.082	2.080	2.042	2.086	2.077
Cu1-P1	2.265	2.257	2.257	2.265	2.254	2.250
Cu1-P2	2.235	2.240	2.240	2.280	2.239	2.239
Cu1-I1	2.649	2.646	2.651	2.706	2.626	2.631
N1-Cu1-P1	98.9	101.2	101.2	105.2	102.3	103.5
N1-Cu1-P2	112.5	110.4	110.4	114.5	110.6	110.6
N1-Cu1-I1	103.0	102.9	102.9	98.2	103.8	104.0
P1-Cu1-I1	103.1	103.2	103.2	100.7	105.6	104.5
P2-Cu1-I1	110.9	110.9	111.0	107.1	108.7	109.3
P2-Cu1-P1	125.8	125.5	125.7	126.5	124.2	123.5
τ_4	0.87	0.88	0.87	0.90	0.90	0.90

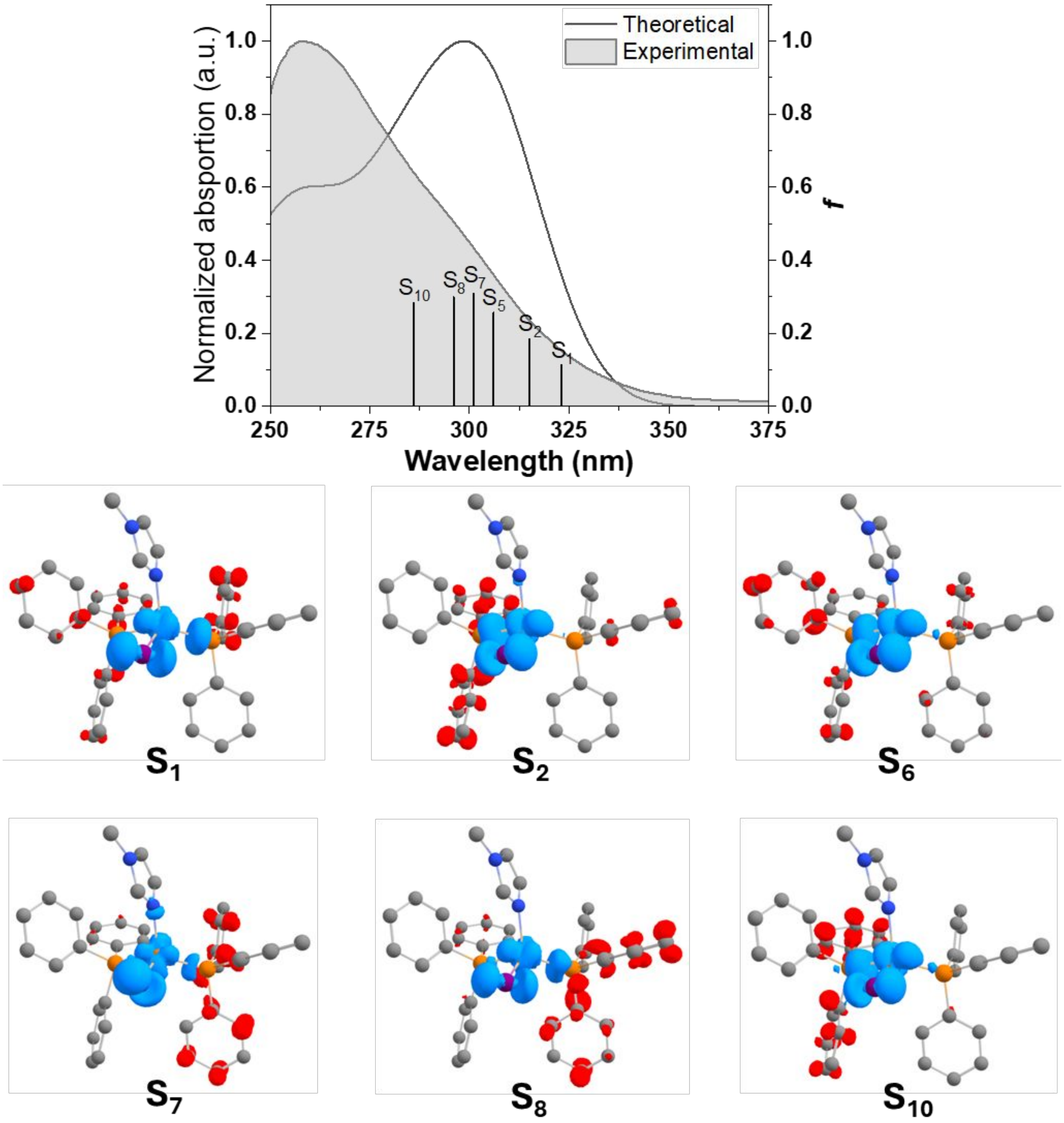

Figure S34 - Comparison between experimental and calculated absorption spectra within ZORA-D3-PBEO/def2-TZVP(-f) theory level for **1**. The six most intense transitions, their respective oscillator strength, and their TD-DFT difference density plots are depicted.

6. X-ray crystallography

Diffracted data were collected with a Kappa APEX II DUO diffractometer, at 150(2) K, with graphite-monochromated Mo Kα radiation (λ = 0.71073 Å). Selected crystallographic data are listed in the tables below. Crystal structures were solved by the dual space method using the SHELXT program and refined by full-matrix least-squares on F^2 using the SHELXL software. Full crystallographic tables (including structure

factors) for complexes have been deposited with the CCDC 2442777 (complex 1), 2443173 (complex 2), 2442778 (complex 3), 2443174 (complex 4), and 2443175 (complex 5).

Table S3 – Crystal data and structure refinement for complex **3**.

Empirical formula	$C_{42}H_{40}CuIN_2P_2$
Formula weight	825.14
Temperature / K	150(2)
Wavelength / Å	0.71073
Crystal system	Monoclinic
Space group	P 2_1/n
Unit cell dimensions	a = 9.3945(5) Å
	b = 19.5349(11) Å
	c = 20.6158(11) Å
	β = 95.870(1)°
Volume / $Å^3$	3763.6(4)
Z	4
Calculated density / Mg/m^3	1.456
Absorption coefficient / mm^{-1}	1.517
F(000)	1672
Crystal size / mm^3	0.300 x 0.180 x 0.060
Theta range for data collection	1.986 to 32.690
Index ranges	-13<=h<=14, -29<=k<=26, -31<=l<=31
Reflections collected	54866
Independent reflections	13798 [R(int) = 0.0293]
Completeness to theta = 25.242°	100.0 %
Absorption correction	Semi-empirical from equivalents
Max. and min. transmission	0.7464 and 0.6735
Refinement method	Full-matrix least-squares on F^2
Data/restrains/parameters	13798 / 0 / 435
Goodness-of-fit on F^2	1.051
Final R indices [I>2sigma(I)]	R1 = 0.0325, wR2 = 0.0596
R indices (all data)	R1 = 0.0506, wR2 = 0.0645
Extinction coefficient	n/a
Largest difference peak and hole / $Å^{-3}$	0.690 and -0.863

Table S4 – Bond lengths and angles for complex **3**.

Length / Å		Angle / °	
Cu(1)-N(1)	2.0424(14)	N(1)-Cu(1)-P(2)	114.51(4)
Cu(1)-P(2)	2.2651(5)	N(1)-Cu(1)-P(1)	105.25(4)
Cu(1)-P(1)	2.2800(5)	P(2)-Cu(1)-P(1)	126.539(18)
Cu(1)-I(1)	2.7065(3)	N(1)-Cu(1)-I(1)	98.18(4)
N(1)-C(1)	1.317(2)	P(2)-Cu(1)-I(1)	107.083(13)
N(1)-C(3)	1.375(2)	P(1)-Cu(1)-I(1)	100.752(13)
C(1)-N(2)	1.346(2)	C(1)-N(1)-C(3)	105.13(15)
C(1)-H(1)	0.9500	C(1)-N(1)-Cu(1)	124.37(12)
N(2)-C(2)	1.374(2)	C(3)-N(1)-Cu(1)	129.69(12)
N(2)-C(4)	1.472(2)	N(1)-C(1)-N(2)	111.78(15)
C(2)-C(3)	1.354(3)	N(1)-C(1)-H(1)	124.1
C(2)-H(2)	0.9500	N(2)-C(1)-H(1)	124.1
C(3)-H(3)	0.9500	C(1)-N(2)-C(2)	107.00(15)
C(4)-C(6)	1.508(3)	C(1)-N(2)-C(4)	125.05(15)
C(4)-C(5)	1.519(3)	C(2)-N(2)-C(4)	127.89(15)
C(4)-H(4)	10.000	C(3)-C(2)-N(2)	105.86(15)
C(5)-H(5A)	0.9800	C(3)-C(2)-H(2)	127.1
C(5)-H(5B)	0.9800	N(2)-C(2)-H(2)	127.1
C(5)-H(5C)	0.9800	C(2)-C(3)-N(1)	110.23(16)
C(6)-H(6A)	0.9800	C(2)-C(3)-H(3)	124.9
C(6)-H(6B)	0.9800	N(1)-C(3)-H(3)	124.9
C(6)-H(6C)	0.9800	N(2)-C(4)-C(6)	110.66(16)
P(1)-C(31)	1.8230(16)	N(2)-C(4)-C(5)	110.01(15)
P(1)-C(21)	1.8331(17)	C(6)-C(4)-C(5)	112.48(17)
P(1)-C(11)	1.8372(17)	N(2)-C(4)-H(4)	107.8
C(11)-C(16)	1.394(2)	C(6)-C(4)-H(4)	107.8

C(11)-C(12)	1.395(2)	C(5)-C(4)-H(4)	107.8
C(12)-C(13)	1.387(3)	C(4)-C(5)-H(5A)	109.5
C(12)-H(12)	0.9500	C(4)-C(5)-H(5B)	109.5
C(13)-C(14)	1.382(3)	H(5A)-C(5)-H(5B)	109.5
C(13)-H(13)	0.9500	C(4)-C(5)-H(5C)	109.5
C(14)-C(15)	1.379(3)	H(5A)-C(5)-H(5C)	109.5
C(14)-H(14)	0.9500	H(5B)-C(5)-H(5C)	109.5
C(15)-C(16)	1.388(3)	C(4)-C(6)-H(6A)	109.5
C(15)-H(15)	0.9500	C(4)-C(6)-H(6B)	109.5
C(16)-H(16)	0.9500	H(6A)-C(6)-H(6B)	109.5
C(21)-C(26)	1.391(2)	C(4)-C(6)-H(6C)	109.5
C(21)-C(22)	1.395(2)	H(6A)-C(6)-H(6C)	109.5
C(22)-C(23)	1.393(3)	H(6B)-C(6)-H(6C)	109.5
C(22)-H(22)	0.9500	C(31)-P(1)-C(21)	102.10(8)
C(23)-C(24)	1.379(3)	C(31)-P(1)-C(11)	105.71(7)
C(23)-H(23)	0.9500	C(21)-P(1)-C(11)	100.60(8)
C(24)-C(25)	1.382(3)	C(31)-P(1)-Cu(1)	110.24(5)
C(24)-H(24)	0.9500	C(21)-P(1)-Cu(1)	122.48(6)
C(25)-C(26)	1.390(3)	C(11)-P(1)-Cu(1)	113.92(6)
C(25)-H(25)	0.9500	C(16)-C(11)-C(12)	118.73(16)
C(26)-H(26)	0.9500	C(16)-C(11)-P(1)	122.28(13)
C(31)-C(32)	1.386(2)	C(12)-C(11)-P(1)	118.87(13)
C(31)-C(36)	1.390(2)	C(13)-C(12)-C(11)	120.26(17)
C(32)-C(33)	1.395(2)	C(13)-C(12)-H(12)	119.9
C(32)-H(32)	0.9500	C(11)-C(12)-H(12)	119.9
C(33)-C(34)	1.377(3)	C(14)-C(13)-C(12)	120.59(18)
C(33)-H(33)	0.9500	C(14)-C(13)-H(13)	119.7
C(34)-C(35)	1.384(3)	C(12)-C(13)-H(13)	119.7
C(34)-H(34)	0.9500	C(15)-C(14)-C(13)	119.47(18)
C(35)-C(36)	1.389(2)	C(15)-C(14)-H(14)	120.3
C(35)-H(35)	0.9500	C(13)-C(14)-H(14)	120.3

C(36)-H(36)	0.9500	C(14)-C(15)-C(16)	120.54(18)
P(2)-C(41)	1.8273(17)	C(14)-C(15)-H(15)	119.7
P(2)-C(51)	1.8275(18)	C(16)-C(15)-H(15)	119.7
P(2)-C(61)	1.8312(18)	C(15)-C(16)-C(11)	120.39(17)
C(41)-C(42)	1.387(2)	C(15)-C(16)-H(16)	119.8
C(41)-C(46)	1.391(2)	C(11)-C(16)-H(16)	119.8
C(42)-C(43)	1.388(3)	C(26)-C(21)-C(22)	118.25(16)
C(42)-H(42)	0.9500	C(26)-C(21)-P(1)	123.49(13)
C(43)-C(44)	1.381(3)	C(22)-C(21)-P(1)	118.22(13)
C(43)-H(43)	0.9500	C(23)-C(22)-C(21)	121.13(18)
C(44)-C(45)	1.380(3)	C(23)-C(22)-H(22)	119.4
C(44)-H(44)	0.9500	C(21)-C(22)-H(22)	119.4
C(45)-C(46)	1.387(3)	C(24)-C(23)-C(22)	119.77(19)
C(45)-H(45)	0.9500	C(24)-C(23)-H(23)	120.1
C(46)-H(46)	0.9500	C(22)-C(23)-H(23)	120.1
C(51)-C(52)	1.381(3)	C(23)-C(24)-C(25)	119.73(18)
C(51)-C(56)	1.390(3)	C(23)-C(24)-H(24)	120.1
C(52)-C(53)	1.396(3)	C(25)-C(24)-H(24)	120.1
C(52)-H(52)	0.9500	C(24)-C(25)-C(26)	120.64(18)
C(53)-C(54)	1.371(4)	C(24)-C(25)-H(25)	119.7
C(53)-H(53)	0.9500	C(26)-C(25)-H(25)	119.7
C(54)-C(55)	1.374(4)	C(25)-C(26)-C(21)	120.47(17)
C(54)-H(54)	0.9500	C(25)-C(26)-H(26)	119.8
C(55)-C(56)	1.386(3)	C(21)-C(26)-H(26)	119.8
C(55)-H(55)	0.9500	C(32)-C(31)-C(36)	119.19(15)
C(56)-H(56)	0.9500	C(32)-C(31)-P(1)	123.89(13)
C(61)-C(62)	1.388(3)	C(36)-C(31)-P(1)	116.90(13)
C(61)-C(66)	1.397(3)	C(31)-C(32)-C(33)	120.02(17)
C(62)-C(63)	1.394(3)	C(31)-C(32)-H(32)	120.0
C(62)-H(62)	0.9500	C(33)-C(32)-H(32)	120.0
C(63)-C(64)	1.379(4)	C(34)-C(33)-C(32)	120.40(18)

C(63)-H(63)	0.9500	C(34)-C(33)-H(33)	119.8
C(64)-C(65)	1.383(3)	C(32)-C(33)-H(33)	119.8
C(64)-H(64)	0.9500	C(33)-C(34)-C(35)	119.94(17)
C(65)-C(66)	1.390(3)	C(33)-C(34)-H(34)	120.0
C(65)-H(65)	0.9500	C(35)-C(34)-H(34)	120.0
C(66)-H(66)	0.9500	C(34)-C(35)-C(36)	119.84(18)
		C(34)-C(35)-H(35)	120.1
		C(36)-C(35)-H(35)	120.1
		C(35)-C(36)-C(31)	120.58(17)
		C(35)-C(36)-H(36)	119.7
		C(31)-C(36)-H(36)	119.7
		C(41)-P(2)-C(51)	102.88(8)
		C(41)-P(2)-C(61)	101.93(8)
		C(51)-P(2)-C(61)	103.75(8)
		C(41)-P(2)-Cu(1)	113.89(6)
		C(51)-P(2)-Cu(1)	117.96(6)
		C(61)-P(2)-Cu(1)	114.48(6)
		C(42)-C(41)-C(46)	118.30(16)
		C(42)-C(41)-P(2)	117.33(13)
		C(46)-C(41)-P(2)	124.37(14)
		C(41)-C(42)-C(43)	121.26(17)
		C(41)-C(42)-H(42)	119.4
		C(43)-C(42)-H(42)	119.4
		C(44)-C(43)-C(42)	119.73(19)
		C(44)-C(43)-H(43)	120.1
		C(42)-C(43)-H(43)	120.1
		C(45)-C(44)-C(43)	119.77(18)
		C(45)-C(44)-H(44)	120.1
		C(43)-C(44)-H(44)	120.1
		C(44)-C(45)-C(46)	120.36(18)
		C(44)-C(45)-H(45)	119.8

		C(46)-C(45)-H(45)	119.8
		C(45)-C(46)-C(41)	120.56(18)
		C(45)-C(46)-H(46)	119.7
		C(41)-C(46)-H(46)	119.7
		C(52)-C(51)-C(56)	118.11(18)
		C(52)-C(51)-P(2)	123.30(15)
		C(56)-C(51)-P(2)	118.53(15)
		C(51)-C(52)-C(53)	120.9(2)
		C(51)-C(52)-H(52)	119.5
		C(53)-C(52)-H(52)	119.5
		C(54)-C(53)-C(52)	120.2(2)
		C(54)-C(53)-H(53)	119.9
		C(52)-C(53)-H(53)	119.9
		C(53)-C(54)-C(55)	119.4(2)
		C(53)-C(54)-H(54)	120.3
		C(55)-C(54)-H(54)	120.3
		C(54)-C(55)-C(56)	120.6(2)
		C(54)-C(55)-H(55)	119.7
		C(56)-C(55)-H(55)	119.7
		C(55)-C(56)-C(51)	120.7(2)
		C(55)-C(56)-H(56)	119.7
		C(51)-C(56)-H(56)	119.7
		C(62)-C(61)-C(66)	118.68(18)
		C(62)-C(61)-P(2)	124.23(15)
		C(66)-C(61)-P(2)	117.08(14)
		C(61)-C(62)-C(63)	120.2(2)
		C(61)-C(62)-H(62)	119.9
		C(63)-C(62)-H(62)	119.9
		C(64)-C(63)-C(62)	120.6(2)
		C(64)-C(63)-H(63)	119.7
		C(62)-C(63)-H(63)	119.7

		C(63)-C(64)-C(65)	119.72(19)
		C(63)-C(64)-H(64)	120.1
		C(65)-C(64)-H(64)	120.1
		C(64)-C(65)-C(66)	120.0(2)
		C(64)-C(65)-H(65)	120.0
		C(66)-C(65)-H(65)	120.0
		C(65)-C(66)-C(61)	120.8(2)
		C(65)-C(66)-H(66)	119.6
		C(61)-C(66)-H(66)	119.6

References

(1) Loozen, H. J. J.; Drouen, J. J. M.; Piepers, O. Thermal Decarboxylation of N-Alkoxycarbonylimidazoles. Improved and Convenient Procedure for N-Alkylation of Imidazoles. *J Org Chem* **1975**, *40* (22), 3279–3280.

(2) Gridnev, A. A.; Mihaltseva, I. M. Synthesis of 1-Alkylimidazoles. *Synth Commun* **1994**, *24* (11), 1547–1555.

(3) Milen, M.; Grün, A.; Bálint, E.; Dancsó, A.; Keglevich, G. Solid–Liquid Phase Alkylation of N-Heterocycles: Microwave-Assisted Synthesis as an Environmentally Friendly Alternative. *Synth Commun* **2010**, *40* (15), 2291–2301.
